# BMP9 Modulates IL-33 Signaling to Mitigate EndMT in Pulmonary Arterial Hypertension

**DOI:** 10.1161/HYPERTENSIONAHA.125.24916

**Published:** 2026-01-22

**Authors:** Clarissa Becher, Esmee J. Groeneveld, Rozenn Quarck, Beau Neep, Xiaoke Pan, Robert Szulcek, Ly Tu, Christophe Guignabert, Harm Jan Bogaard, Paul B. Yu, Frances de Man, Gonzalo Sanchez-Duffhues, Marie-José Goumans

**Affiliations:** Department of Cell and Chemical Biology, Leiden University Medical Center, the Netherlands (C.B., E.J.G., R.S., G.S.-D., M.-J.G.).; Department of Chronic Diseases and Metabolism, Laboratory of Respiratory Diseases and Thoracic Surgery, KU Leuven-University of Leuven, Belgium (R.Q.).; Department of Pulmonary Medicine, PHEniX Laboratory, Amsterdam University Medical Center (Vrije Universiteit), The Netherlands (B.N., X.P., R.S., H.J.B., F.d.M.).; Amsterdam Cardiovascular Sciences, Pulmonary Hypertension and Thrombosis, Amsterdam, the Netherlands (B.N., X.P., H.J.B., F.d.M.).; Laboratory of In Vitro Modeling Systems of Pulmonary and Thrombotic Diseases, Institute of Physiology, Charité-Universitätsmedizin Berlin, Germany (R.S.).; Faculté de Médecine, Hypertension Pulmonaire: Physiopathology and Innovation Thérapeutique, Université Paris-Saclay, Le Kremlin-Bicêtre, France (L.T., C.G.).; Division of Cardiovascular Medicine, Department of Medicine, Brigham and Women’s Hospital, Harvard Medical School, Boston, MA (P.B.Y.).; Nanomaterials and Nanotechnology Research Center (CINN-CSIC), Health Research Institute of Asturias (ISPA), Oviedo, Spain (G.S.-D.).

**Keywords:** bone morphogenetic proteins, cytokines, disease models, animal, endothelial cells, pulmonary arterial hypertension

## Abstract

**BACKGROUND:**

Pulmonary arterial hypertension (PAH) is a progressive disorder involving disrupted BMP (bone morphogenetic protein) signaling, pulmonary inflammation, and endothelial-to-mesenchymal transition (EndMT). We hypothesized that IL (interleukin)-33 signaling contributes to PAH progression by inducing EndMT and interacting with BMP9, a key modulator of inflammation and vascular remodeling.

**METHODS:**

IL-33 expression was assessed in lung tissues from Sugen/hypoxia and control mice, as well as in pulmonary arterial endothelial cells (PAECs) and lung tissues from patients with PAH and healthy donors. EndMT and signaling pathways were analyzed in PAECs and microvascular endothelial cells (MVECs) exposed to IL-33, BMP9, and sST2 (soluble supression of tumorigenicity 2) using quantitative polymerase chain reaction, Western blotting, ELISA, and immunostaining. Plasma BMP9 and sST2 levels were quantified in patients with PAH.

**RESULTS:**

Immunofluorescent analysis revealed elevated IL-33 expression in pulmonary endothelial cells of Sugen/hypoxia mice compared with controls, consistent with findings in PAECs from patients with PAH. BMP9 significantly upregulated sST2 expression in human PAEC and microvascular endothelial cells, inhibited IL-33 target gene expression, and effectively suppressed IL-33-induced EndMT. Notably, BMP9 demonstrated greater efficacy in preventing EndMT compared with rsST2 (recombinant soluble ST2) or ST2L-neutralizing antibodies. Circulating BMP9 and sST2 levels in the plasma of patients with PAH were positively correlated in specific patient groups stratified by sex, age, and New York Heart Association functional class, suggesting a protective role of BMP9 in modulating IL-33-induced EndMT.

**CONCLUSIONS:**

BMP9 plays a protective role against IL-33-induced EndMT in PAECs by upregulating sST2 expression and neutralizing IL-33, suggesting that targeting the IL-33 signaling pathway may represent a promising therapeutic strategy to mitigate EndMT in PAH.

**NOVELTY AND RELEVANCE**

**What Is New?**

This study identifies IL (interleukin)-33 as a previously underrecognized mediator of endothelial-to-mesenchymal transition in pulmonary arterial hypertension. We demonstrate for the first time that BMP (bone morphogenetic protein) 9 counters IL-33-induced endothelial-to-mesenchymal transition by upregulating sST2 (soluble supression of tumorigenicity 2), which functions as a decoy receptor to suppress IL-33 signaling.

**What Is Relevant?**

Both IL-33 and sST2 levels are altered in pulmonary arterial hypertension, with IL-33 upregulated in pulmonary endothelium and sST2 elevated in the circulation of patients with more severe disease. The identification of BMP9 as a driver of sST2 offers new mechanistic insight into how protective signaling pathways can modulate inflammatory drivers of vascular remodeling.

**Clinical/Pathophysiological Implications?**

Targeting IL-33 signaling may represent a novel therapeutic strategy to mitigate endothelial dysfunction and vascular remodeling in pulmonary arterial hypertension. While BMP9 has been proposed as a therapy for restoring BMPR2 signaling, its paradoxical effects necessitate caution. IL-33 inhibition, potentially via clinically available monoclonal antibodies, may provide a more focused approach to preventing endothelial-to-mesenchymal transition and disease progression. sST2 may also serve as a biomarker of disease activity and response to therapy in future clinical applications.

**See Jankauskas et al**

Pulmonary arterial hypertension (PAH) is a severe and rare disorder defined as elevated pulmonary arterial pressure (>20 mm Hg) and pulmonary vascular resistance (>2 Wood units). PAH is characterized by progressive occlusive remodeling of the distal pulmonary vasculature, resulting in increased pulmonary artery pressure, right ventricular dysfunction, right ventricular failure, and death if left untreated.^1^ Key pathological processes include the dysregulated proliferation of endothelial cells (ECs) and smooth muscle cells, inflammation, apoptosis, and thrombosis. Structural abnormalities in the pulmonary arteries, such as medial and intimal thickening, capillary rarefaction, and the formation of disorganized plexiform lesions, are hallmarks of PAH.^2^ The defining feature of these changes is the elevated presence of cells expressing α-SMA (α-smooth muscle actin), indicative of a transition of ECs into mesenchymal-like cells, contributing to vascular remodeling. This process, known as endothelial-to-mesenchymal transition (EndMT), involves the loss of endothelial markers, such as CD31 and VE-cadherin (vascular endothelial cadherin), with simultaneous acquisition of mesenchymal markers including α-SMA, transgelin (SM22α [smooth muscle protein 22-alpha]), and fibronectin. EndMT disrupts vascular barrier function, cell-cell adhesion, cell migration, and immune cell infiltration, thereby playing a pivotal role in the vascular remodeling associated with PAH.^3^ Mutations in the *BMPR2* gene are frequently implicated in PAH pathogenesis. Combined with inflammatory cues, these mutations are thought to suppress BMPR2 (bone morphogenetic protein R2) expression while exacerbating pathological transforming growth factor-β (TGF-β) signaling.^4^ In patients with PAH, circulating TGF-β1 and activin A levels are elevated.^5,6^ This imbalance has prompted the exploration of therapeutic strategies targeting the TGF-β pathway, such as sotatercept, and approaches aimed at enhancing BMP9 activity have been explored.^7^ However, the role of BMP9 in PAH remains controversial. While some studies suggest that enhancing BMP9 is beneficial, others report that genetic deletion or inhibition of BMP9 mitigates the onset and progression of PAH.^8^ BMP9 is recognized to be crucial for cardiovascular homeostasis and implicated in the development of pulmonary hypertension (PH), with its role appearing context- and time-dependent, particularly in the pulmonary endothelium. Congruently, the impact of BMP9 in the vasculature is strongly influenced by inflammatory responses.^9^ IL (interleukin)-33, part of the IL-1 cytokine family, is produced by ECs during cellular stress or tissue injury and functions as an alarmin.^10^ IL-33 signals through its unique receptor ST2 (supression of tumorigenicity 2), forming a complex with IL-1 receptor accessory protein (IL-1RAcP or IL1RAP). This signaling cascade activates pathways such as MAPKs (mitogen-activated protein kinases) and NF-κB (nuclear factor-κB) mediated by the adaptor protein MyD88 (myeloid differentiation primary response protein 88).^11^ The sST2 (soluble form of ST2) functions as a decoy receptor, effectively neutralizing IL-33 activity. Aberrant IL-33 signaling has been implicated in various lung diseases, including chronic obstructive pulmonary disease, asthma, and allergic and airway inflammatory disorders.^12^ In a murine model of hypoxia-induced PH, IL-33 has been shown to exacerbate vascular remodeling under hypoxic conditions.^13^ In addition, IL-33 signaling stimulates the proliferation of control pulmonary arterial EC (PAEC), while blockade of the membrane-bound ST2 receptor (ST2L) reduces these effects in a Sugen/hypoxia mouse model.^14^

We hypothesized that altered pulmonary vascular IL-33 expression would perpetuate PAH progression through the induction of EndMT and interaction with the BMP9 signaling pathway. Using primary PAECs and MVECs from patients with PAH, experimental animal models, and patient cohorts, we unveil a new crosstalk between IL-33 and BMP9 in the regulation of EndMT in PAH. Specifically, BMP9 was shown to inhibit EndMT in pulmonary ECs by upregulating sST2. In patient cohorts, we observed elevated sST2 levels in men, individuals aged >67 years, and patients with more severe PAH. A positive correlation was observed between sST2 and BMP9 levels within these stratified groups, highlighting their potential interplay in PAH pathogenesis.

## Methods

### Data Availability

The data that support the findings of this study are available from the corresponding author upon reasonable request.

### Reagents

Human recombinant BMP4 (314-BP-010/CF), BMP6 (507-BP-020/CF), BMP9 (3209-BP-010/CF), BMP10 (2926-BP-025/CF), activin A (338-AC-010/CF), TGF-b1 (240-B-010/CF), and recombinant human soluble ST2/IL-33R Fc (523-ST-100) were obtained from R&D Systems. Human recombinant IL-33 (CYT-425) was purchased from Prospec, and LDN-193189 (6053), a selective BMP signaling inhibitor targeting ALK1/2/3 and 6 (Activin Receptor-Like Kinase 1/2/3 and 6) other kinases, was purchased from Tocris (Table S1). Carrier-free ligands were reconstituted in 4-mmol/L HCl and 0.1% BSA.

### Blood Samples

Patients diagnosed with idiopathic PAH and hereditary PAH, according to the European Respiratory Society and European Society of Cardiology guidelines, who underwent right heart catheterization for diagnosis purposes at the University Hospital Leuven (Belgium) between 2009 and December 2023, were included.^1^ Blood samples were collected on ethylenediaminetetraacetic acid at the time of diagnostic right heart catheterization, and plasma was prepared as previously described.^15^ The study protocol was approved by the Institutional Ethics Committee of the University Hospital Leuven, and all participants gave written informed consent.

### Cell Culture

PAEC and MVEC were isolated from healthy controls or patients with PAH. PAH cells were obtained from lung tissue during transplantation, while control cells were derived from noncancerous lung tissue dissected from pneumonectomy patients. Patient characteristics of the cells are displayed in Table S2. The study was approved by the VU University Medical Center ethics board (protocol-nr: 2012/306, non-WMO [Wet Medisch-wetenschappelijk Onderzoek met mensen]) and performed as previously described.^16^ Both cell types were cultured on 0.1% (w/v) gelatin-coated (Sigma-Aldrich, G1890) culture ware (Corning) in complete EC medium (ScienCell, 1001) supplemented with 100-U/mL Pen/Strep, 1% EC growth supplement, and 5% FCS. Cells were maintained at 37 °C and 5% CO₂ in a humidified atmosphere and regularly tested for mycoplasma.

### EndMT Assay

PAECs from patients with PAH or healthy donors were cultured in Lan-Tek II chamber slides (Thermo Fisher, 154534) with ECM complete medium. At 80% confluence, cells were treated with BMP9 (1 ng/mL, 3 hours), rsST2 (recombinant soluble ST2; 1 μg/mL), ST2 neutralizing antibody (1 μg/mL), or vehicle control (30 minutes), followed by IL-33 (100 ng/mL, 72 hours). Subsequent fixation, immunostaining, and image acquisition were performed, as described in the Supplemental Methods. Negative control stainings were performed, and representative images are provided in Figure S6 to confirm antibody specificity.

### siRNA Experiments

PAECs were transfected with 25-nM ON-TARGETplus SMARTpool siRNAs targeting ALK1 (L-005302-02-0005, Dharmacon), ENG (endoglin; L-011026-00-0005, Dharmacon), or nontargeting control siRNA (siScr, D-001810-10-20; Dharmacon) using the manufacturer’s protocol in antibiotic-free medium. After 24 hours, the medium was replaced with standard culture medium containing FCS and penicillin/streptomycin for an additional 24 hours. Cells were then starved overnight and stimulated with BMP9 (1 ng/mL) for 3 hours before RNA isolation for quantitative polymerase chain reaction analysis.

### Reverse Transcription-Quantitative Polymerase Chain Reaction

Cells were cultured in 12-well plates with ECM complete medium until 80% confluence and then starved (6 hours) in ECM basal medium with 0.1% FBS. Cells were incubated (16 hours) with TGF-β1 (1 ng/mL), activin A (50 ng/mL), or vehicle control. Alternatively, they were treated (3 hours) with BMP4, BMP6 (50 ng/mL each), BMP9, BMP10 (1 ng/mL each), or ligand buffer. The different stimulation times reflect the faster signaling kinetics of BMPs compared with the slower response of TGF-β ligands in ECs. For inhibition assays, cells were pretreated (30 minutes) with LDN-193189 (120 nmol/L) or dimethyl sulfoxide, followed by BMP9 (1 ng/mL, 3 hours). RNA was extracted using the RNA Miniprep System (Promega, Z6012) after PBS washes. cDNA synthesis was performed with 500-ng RNA using the RevertAid First Strand cDNA Synthesis Kit (Thermo Fisher, K1632). Reverse transcription-quantitative polymerase chain reaction was conducted with GoTaq SYBR Green Supermix (Promega, A6001) on a CFX384 Connect Real-Time PCR System (Bio-Rad) per manufacturer’s protocol. Ct values were normalized to Glyceraldehyde-3-phosphate dehydrogenase) GAPDH and Actin Related Protien (ARP) using the ΔΔCt method. Primer sequences and specificities are listed in Table S4. To distinguish between the isoforms of Interleukin-1 Recpetor-Like 1 (IL1RL1), isoform-specific primers were designed. For sST2, the forward primer was placed within the unique exon 1, corresponding to transcript NM_003856.4, ensuring specificity for the soluble isoform. For ST2L, transcript NM_016232.5 served as the reference, and primers were selected from regions absent in the sST2 transcript. Primer specificity was verified using NCBI Primer-BLAST against the respective transcript FASTA sequences.

### Western Blotting

Cells were cultured in 12-well plates with ECM complete medium until confluence and then starved (6 hours) in ECM basal medium with 0.1% FBS. Cells were stimulated with BMP9 (1 ng/mL) or ligand buffer (16 hours). Where indicated, cells were pretreated with rsST2 (1 μg/mL, 30 minutes) before IL-33 (100 ng/mL) stimulation for 5, 15, or 60 minutes. Lysates were subsequently obtained and processed as indicated in the Supplemental Material. All original blots are provided in the Supplemental Material.

### ELISA

Cells were cultured in 24-well plates with ECM complete medium until 80% confluence and then starved for 16 hours. After preincubation with BMP9 (1 ng/mL, 3 hours), IL-33 (100 ng/mL) or vehicle control was added for 24 hours. Supernatants were collected, centrifuged (1000*g*, 20 minutes, 4 °C), and used for protein detection assays. Cytokine and sST2 levels were measured using human IL-33 DuoSET ELISA (R&D Systems, DY3625B) and human sST2 ELISA (Elabscience, E-EL-H6082) per manufacturer’s instructions. sST2 and BMP9 levels in the plasma of patients with PAH were quantified using ELISA kits from Elabscience (E-EL-H6082) and R&D Systems (DY3209), respectively.

### Immunohistochemical Staining

Mouse lung slices were prepared as described previously.^17^ Briefly, 6-month-old male C57 Black 6J mice received weekly subcutaneous SU-5416 (20-mg/kg) injections and were placed in 10% O₂ hypoxia for 3 weeks; a separate cohort remained in normoxia. Human lung tissues were obtained postautopsy from patients with PAH and control subjects without cardiopulmonary abnormalities. Immunohistochemical staining and image analysis of lung sections were performed, as detailed in the Supplemental Methods. Representative images of negative control stainings were performed to confirm antibody specificity (Figure S6).

### Statistical Analysis

Each cell culture experiment was independently replicated at least 3×. In all figure legends, n refers to the number of independent biological replicates (different donors or animals). Technical replicates are indicated as technical replicates where applicable. Statistical analyses and graphing were performed using GraphPad Prism, version 9 (GraphPad Software). Data were analyzed using the Student *t* tests or 1-/2-way ANOVAs with the Tukey post hoc tests for multiple comparisons, as detailed in figure legends. Normality was assessed using the Shapiro-Wilk test. Nonnormally distributed values were log-transformed and expressed as medians with 95% CIs. If log-transformed data were normally distributed, comparisons were made using parametric *t* tests; otherwise, the Mann-Whitney *U* test was used. Pearson and Spearman correlations were applied to normally and nonnormally distributed variables, respectively. *P*<0.05 was considered statistically significant.

## Results

### IL-33 Expression Is Increased In Human PAEC From Patients With PAH and in Pulmonary Vessels of Sugen/Hypoxia Mice

**Figure 1.**
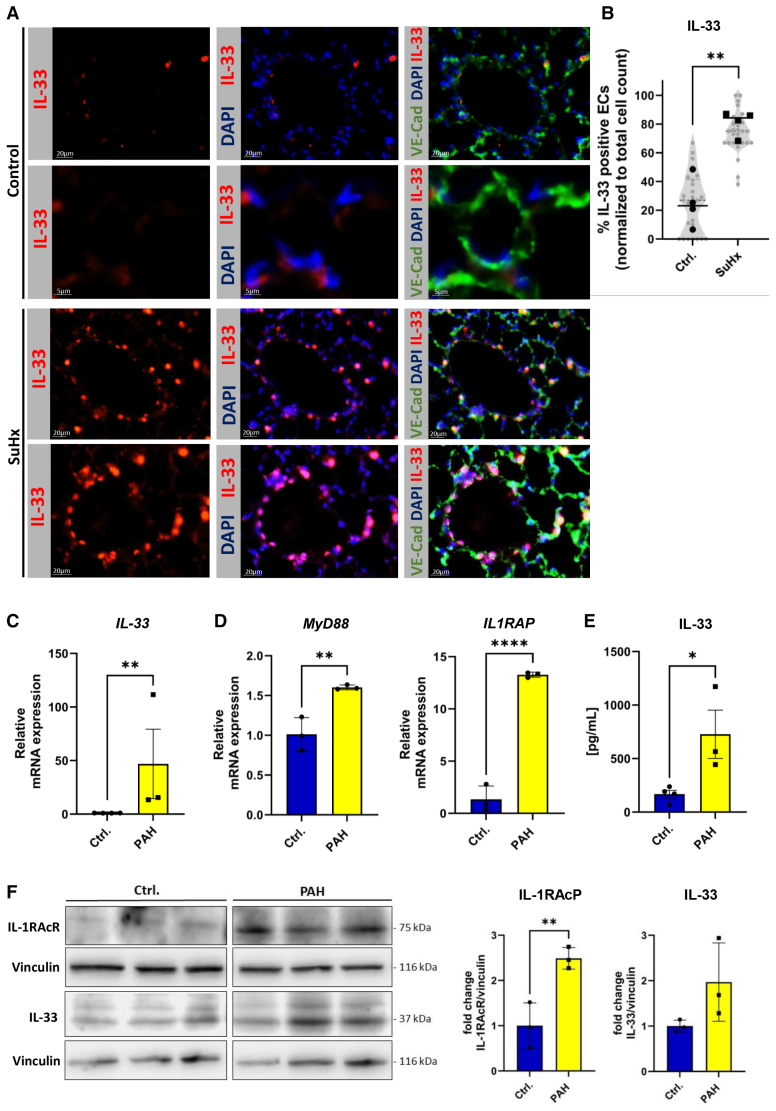
**IL (interleukin)-33 expression is increased in human pulmonary arterial endothelial cells (PAECs) from patients with pulmonary arterial hypertension (PAH) in vitro and in pulmonary vessels of Sugen/hypoxia (Su/Hx) mice in vivo. A**, Representative immunohistochemical images of IL-33, VE-cadherin (vascular endothelial cadherin), and 4′,6-diamidino-2-phenylindole (DAPI) in lung sections from normoxic and Su/Hx-treated mice (n=4). **B**, Quantification of IL-33 and VE-cadherin coexpression in >25 pulmonary vessels. Gray data points represent individual vessels; black data points show the average per animal. **C** and **D**, *IL-33*, *MyD88*, and *IL1RAP* gene expressions in PAH PAEC compared with healthy controls (n=3). **E**, Soluble IL-33 levels in PAH PAEC supernatants vs controls (n=3). **F**, Representative immunoblot and quantification of IL-33 and IL-1RAcR expression in PAECs from patients with PAH (n=3, each analyzed with technical replicates [t.n.]=3 compared with control PAECs [n=3]). Statistical analysis: unpaired Student *t* test; *P*<0.05, *P<0.01, and ***P<0.0001. Data are shown as mean±SD.

IL-33 expression was assessed in lung sections from Sugen/hypoxia and control mice. Immunostaining for VE-cadherin, DAPI (4′,6-diamidino-2-phenylindole), and IL-33 showed significantly increased IL-33 (red) in ECs of pulmonary vessels in Sugen/hypoxia mice compared with normoxic controls, confirming IL-33 upregulation under hypoxia (Figure 1A and 1B). To explore IL-33’s role in PAH pathophysiology, its expression was evaluated in human PAECs. *IL-33* mRNA and secreted IL-33 protein levels were significantly elevated in PAH-derived PAECs compared with controls (Figure 1C and 1E). Analysis of IL-33 downstream signaling revealed increased *MYD88* and *IL1RAP* mRNA expression in PAH PAECs (Figure 1D). Western blot analysis further confirmed upregulated IL-33 and its receptor accessory protein IL-1RAcP, reinforcing the transcriptional data and supporting IL-33 pathway activation in PAH (Figure 1F). These findings indicate enhanced activation of IL-33 signaling in the context of PAH.

### BMP9 Protects From IL-33-Induced EndMT and Induces sST2 Expression in PAEC

**Figure 2.**
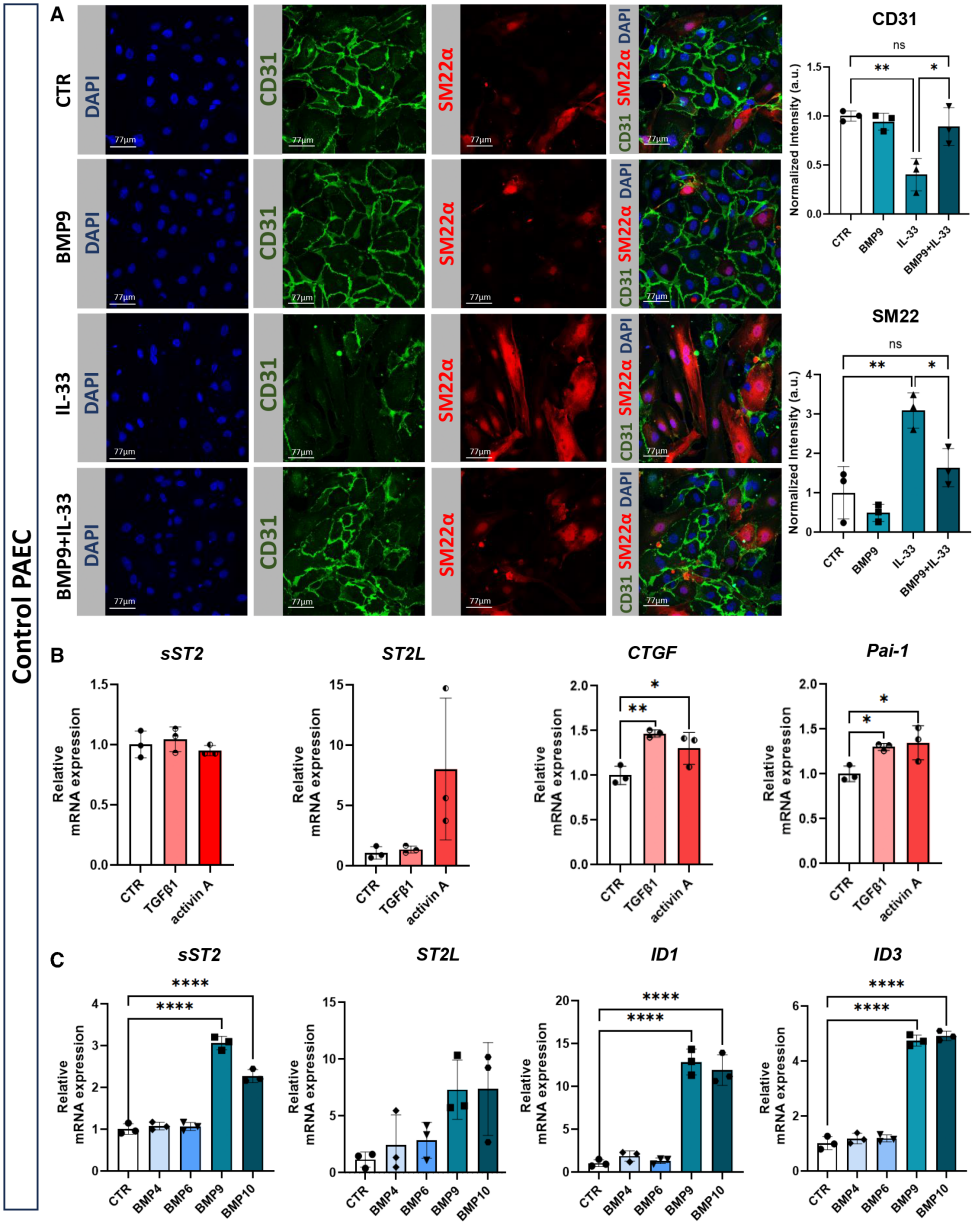
**BMP (bone morphogenetic protein) 9 protects from IL (interleukin)-33–induced endothelial-to-mesenchymal transition (EndMT) and induces sST2 (soluble supression of tumorigenicity 2) expression in control pulmonary arterial endothelial cells (PAECs) in vitro. A**, Representative immunofluorescent staining of PAEC for CD31 (endothelial marker), SM22α (smooth muscle protein 22-alpha; mesenchymal marker), and 4′,6-diamidino-2-phenylindole (DAPI; nuclei). Cells were treated with BMP9 (1 ng/mL), IL-33 (100 ng/mL), both, or left untreated (control [CTR]) for 3 days. Bar graphs show CD31 and SM22α intensity quantification (n=3). **B**, *sST2*, *ST2L*, *CTGF*, and *PAI-1* gene expressions in PAECs after 16-hour stimulation with TGF (transforming growth factor)-β (1 ng/mL), activin A (50 ng/mL), or untreated (CTR; technical replicates [t.n.]=3). **C**, *sST2*, *ST2L*, *ID1*, and *ID3* gene expressions in PAECs after 3-hour stimulation with BMP4 (50 ng/mL), BMP6 (50 ng/mL), BMP9 (1 ng/mL), BMP10 (1 ng/mL), or untreated (CTR; t.n.=3). Statistical analysis: 1-way ANOVA with the Tukey post hoc test; *P<0.05, **P<0.01, and ****P<0.0001. Data are shown as mean±SD.

EndMT is a key feature of endothelial dysfunction in PAH. While IL-6 primes PAH MVECs for EndMT under prolonged BMP9 exposure,^8^ IL-33 also induces EndMT during tissue regeneration.^9,18^ We investigated whether IL-33 alone is sufficient to induce EndMT in PAECs or if BMP9 modulates this response. BMP9 alone had no phenotypic effects on control PAECs, but IL-33 stimulation significantly reduced endothelial marker CD31 and increased mesenchymal marker SM22α, indicating EndMT. However, BMP9 cotreatment maintained a quiescent endothelial phenotype, preventing IL-33-induced EndMT (Figure 2A). Given BMP9’s protective role, we next examined its interaction with the IL-33/ST2 axis and TGF-β/BMP signaling pathways. As TGF-β ligands typically exhibit slower signaling kinetics than BMPs, a 16-hour stimulation, commonly used to assess TGF-β responses in ECs, was applied. To allow direct comparison with the more rapid kinetics of BMP signaling, we additionally performed 3-hour stimulations with TGF-β1 and activin A. In both cases, TGF-β1 and activin A did not alter sST2 expression, whereas BMP9 and BMP10 significantly upregulated sST2, indicating a specific regulatory effect (Figure 2B; Figure S1). Consistently, BMP9 and BMP10 also strongly induced the canonical BMP target genes *ID1* and *ID3* (Figure 2C), confirming their activity under these conditions.

### BMP9 Induces sST2 Expression via ALK1 Signaling in a Dose- and Time-Dependent Manner

**Figure 3.**
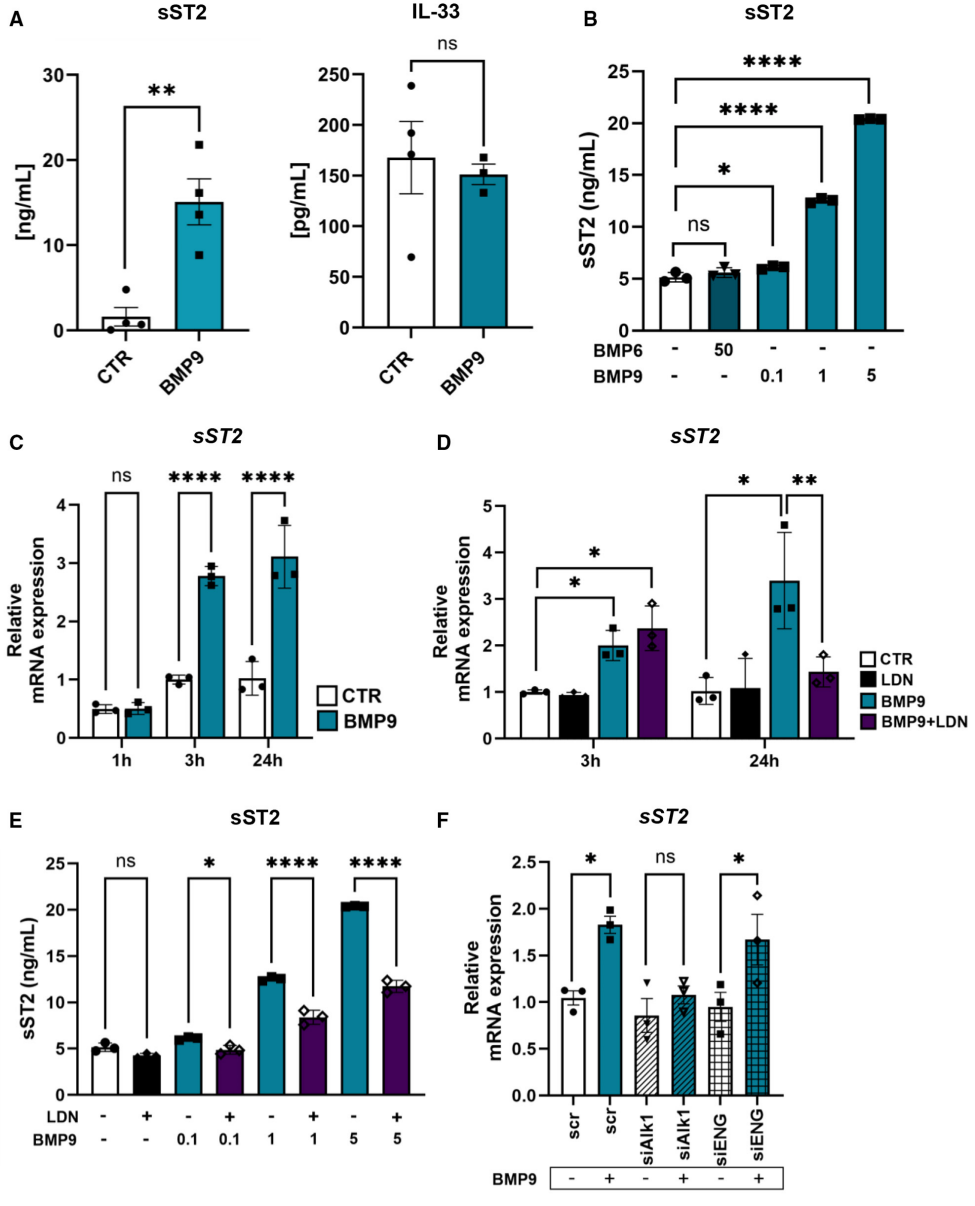
**BMP (bone morphogenetic protein) 9 induces sST2 (soluble supression of tumorigenicity 2) expression in vitro in pulmonary arterial endothelial cells (PAECs) via ALK1 (Activin Receptor-like Kinase) signaling in a dose- and time-dependent manner. A**, Secreted protein levels of sST2 and IL (interleukin)-33 in PAEC supernatants after 24-hour BMP9 (1-ng/mL) stimulation compared with unstimulated controls. Each data point represents 3 biological replicates per donor (n=4). **B**, Dose-dependent increase in sST2 protein secretion following 24-hour stimulation with 0.1-, 1-, or 5-ng/mL BMP9 (technical replicates [t.n.]=3). **C**, Time-dependent induction of sST2 mRNA expression after 1, 3, or 24 hours of BMP9 stimulation (1 ng/mL; t.n.=3). **D**, sST2 mRNA expression in PAECs pretreated with LDN-193189 (120 nmol/L, 30 minutes) followed by BMP9 (1-ng/mL) stimulation for 3 or 24 hours (t.n.=3). **E**, Secreted sST2 protein levels following 24-hour stimulation with increasing concentrations of BMP9 in the presence or absence of LDN-193189 (120 nmol/L; t.n.=3). **F**, sST2 mRNA expression in PAECs transfected with siRNA targeting ALK1 or ENG (endoglin) and stimulated with BMP9 (1 ng/mL, 3 hours; t.n.=3). Statistical analysis: (**A**) unpaired Student *t* test, (**B**, **E**, and **F**) 1-way ANOVA with the Tukey post hoc test for multiple comparisons, and (**C** and **D**) 2-way ANOVA with the Tukey post hoc test for multiple comparisons. *P<0.05, **P<0.01, ***P<0.001, and ****P<0.0001. Data are shown as (**A**) mean±SEM and (**B**–**F**) mean±SD. ns indicates not significant.

Given the strong BMP9-induced sST2 upregulation in PAECs (Figure 2C), we further characterized this effect across multiple donors. BMP9 significantly increased both *sST2* mRNA and secreted protein levels, without affecting *IL-33* mRNA expression or secretion (Figure 3A; Figure S2A). This induction was dose-dependent, with minimal upregulation observed at 0.1-ng/mL BMP9 and a gradual increase at 1 and 5 ng/mL on both mRNA and protein levels (Figure 3B; Figure S2B). Time-course analysis revealed no induction after 1-hour stimulation with 1-ng/mL BMP9, while robust sST2 expression was evident at 3 and 24 hours (Figure 3C), indicating a temporally regulated response. In ECs, BMP9 displays a high affinity for ALK1, which forms a signaling complex with the type II receptor BMPR2.^19^ Therefore, we tested whether ALK1 activity mediates BMP9-induced sST2 expression. Pretreatment with a selective kinase inhibitor, LDN-193189 (120 nmol/L), significantly reduced the transcriptional activation of *ID1* and *ID3* but did not affect BMP9-induced sST2 upregulation at 3 hours. At 24 hours, however, LDN significantly reduced sST2 transcript levels (Figure 3D). As expected, *ID1* expression was minimal at 24 hours due to its early response kinetics (Figure S2D). Importantly, LDN also attenuated BMP9-induced sST2 protein secretion and mRNA expression in a concentration-independent manner (Figure 3E; Figure S2E). LDN-193189 is a broad ATP-competitive inhibitor that blocks several BMP/TGF-β type I receptors, including ALK1, ALK2, ALK3, and ALK6, but its effectiveness can vary depending on the cell type. To directly test which receptor mediates BMP9-induced sST2 expression, we performed siRNA knockdown of *ALK1* and *ENG*. Silencing *ALK1* completely blocked *sST2* mRNA induction by BMP9 at 3 hours, whereas knockdown of *ENG* had no effect (Figure 3F). The efficiency of *ALK1* and *ENG* knockdown was confirmed by quantitative polymerase chain reaction, and siALK1 also inhibited BMP9 target gene expression (Figure S2F). These results confirm that BMP9 induces sST2 expression specifically through ALK1 and independently of ENG.

### BMP9 Prevents IL-33-Induced EndMT Equally Effective as Recombinant Soluble ST2 and Inhibits IL-33 Target Gene Expression

**Figure 4.**
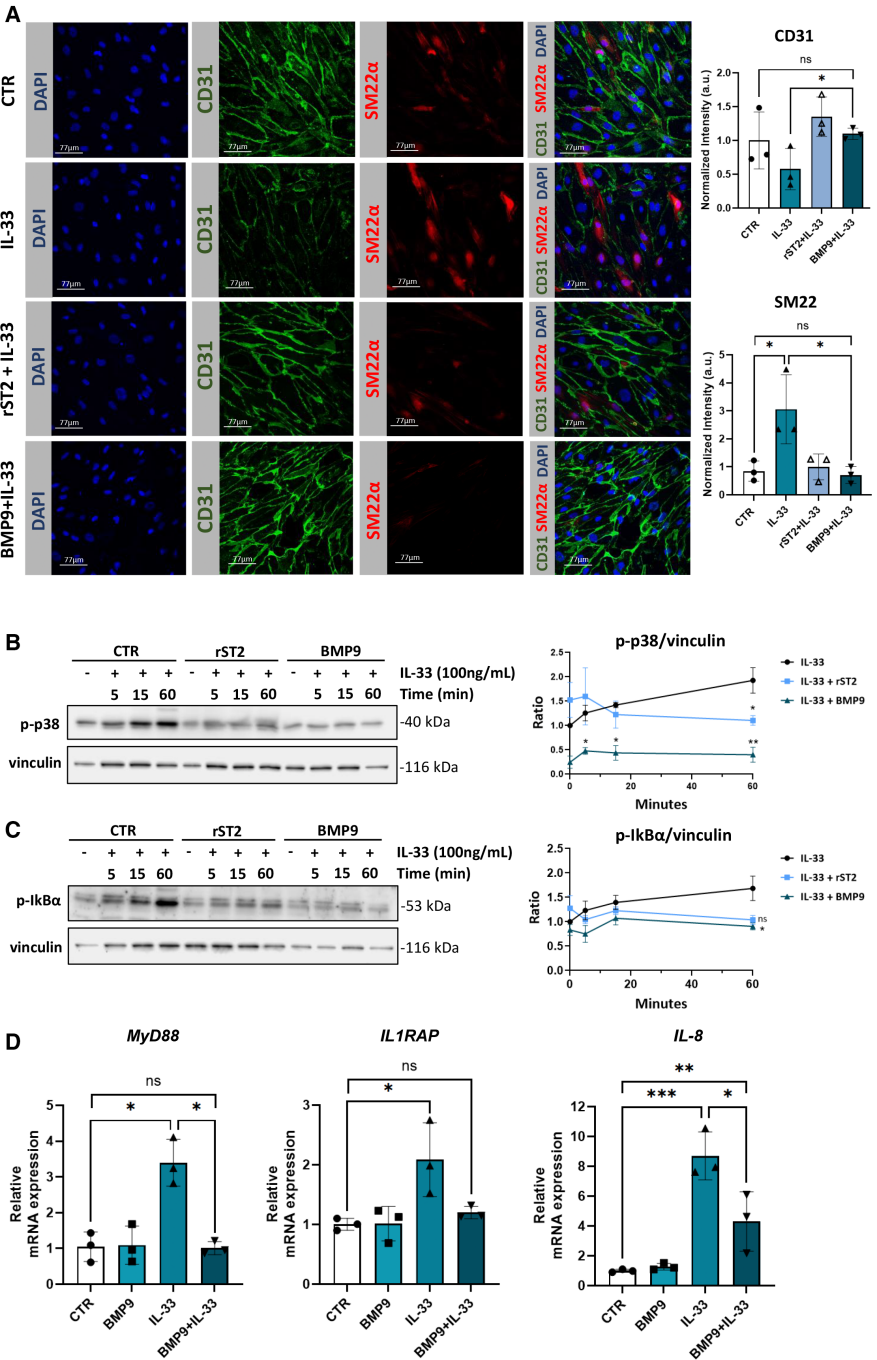
**BMP (bone morphogenetic protein) 9 prevents IL (interleukin)-33–induced endothelial-to-mesenchymal transition (EndMT) in pulmonary arterial endothelial cells (PAECs) in vitro, similar to rsST2 (recombinant soluble ST2), thereby inhibiting IL-33 target gene expression. A**, Representative immunofluorescent staining of for CD31 (endothelial marker), SM22α (smooth muscle protein 22-alpha; mesenchymal marker), and 4′,6-diamidino-2-phenylindole (DAPI; nuclei). Cells were pretreated with BMP9 (1 ng/mL, 3 hours) or rsST2 (1 μg/mL, 30 minutes) before IL-33 (100-ng/mL) stimulation for 3 days. Bar graphs show CD31 and SM22α intensity quantification (n=3). **B** and **C**, Representative immunoblots of p-p38 (phosphorylated p38 mitogen-activated protein kinase) and p-IκBα (phosphorylated inhibitor of nuclear factor kappa-B alpha) in PAECs pretreated overnight with BMP9 (1 ng/mL), rST2 (1 μg/mL, 30 minutes), or left untreated before IL-33 (100-ng/mL) stimulation for 5, 15, or 60 minutes. Densitometric analysis was performed using ImageJ, with protein levels normalized to vinculin and expressed as fold change relative to the 0-minute control (n=3). **D**, *MyD88*, *IL1RAP*, and *IL-8* gene expressions in PAEC pretreated with BMP9 (1 ng/mL, 3 hours) before IL-33 (100 ng/mL, 24 hours) stimulation or left untreated (control [CTR]; n=3). Statistical analysis: 1-way ANOVA (**A** and **D**) or 2-way ANOVA (**B** and **C**) with the Tukey post hoc test; *P*<0.05, *P<0.01, and **P<0.001. Data are shown as mean±SD (**A** and **D**) or mean±SEM (**B** and **C**). ns indicates not significant.

Since BMP9 upregulates sST2 (Figure 3A and 3C), we examined whether this mechanism mediates BMP9’s protection against IL-33-induced EndMT. Immunofluorescent staining confirmed that IL-33 reduced CD31 and increased SM22α expression, indicating EndMT, while pretreatment with BMP9 or rsST2 prevented these changes, preserving endothelial integrity (Figure 4A). Notably, BMP9 was more effective than anti-ST2L antibody treatment in preventing IL-33-induced EndMT, as indicated by better-preserved VE-cadherin expression (Figure S3). To investigate whether BMP9’s protection extends beyond sST2 induction, we analyzed IL-33 downstream signaling. Western Blot showed that IL-33 treatment led to a time-dependent increase in phosphorylated p38 Mitogen-Activated Protein Kinase (p-p38) and phosphorylated Inhibitor of Nuclear Factor Kappa-B Alpha (p-IκBα), indicating pathway activation. While rsST2 slightly reduced their phosphorylation, BMP9 nearly abolished their IL-33-induced activation (Figure 4B and 4C). In parallel, BMP9 significantly reduced IL-33 target genes expression (*MyD88*, *IL1RAP*, and *IL-8*) within 3 hours of treatment (Figure 4D). Thus, BMP9 not only induces sST2 expression but also suppresses IL-33 signaling, further protecting PAECs from IL-33-induced EndMT.

### BMP9 Protects From IL-33-Induced EndMT and Induces sST2 Expression in PAEC From Patients With PAH

**Figure 5.**
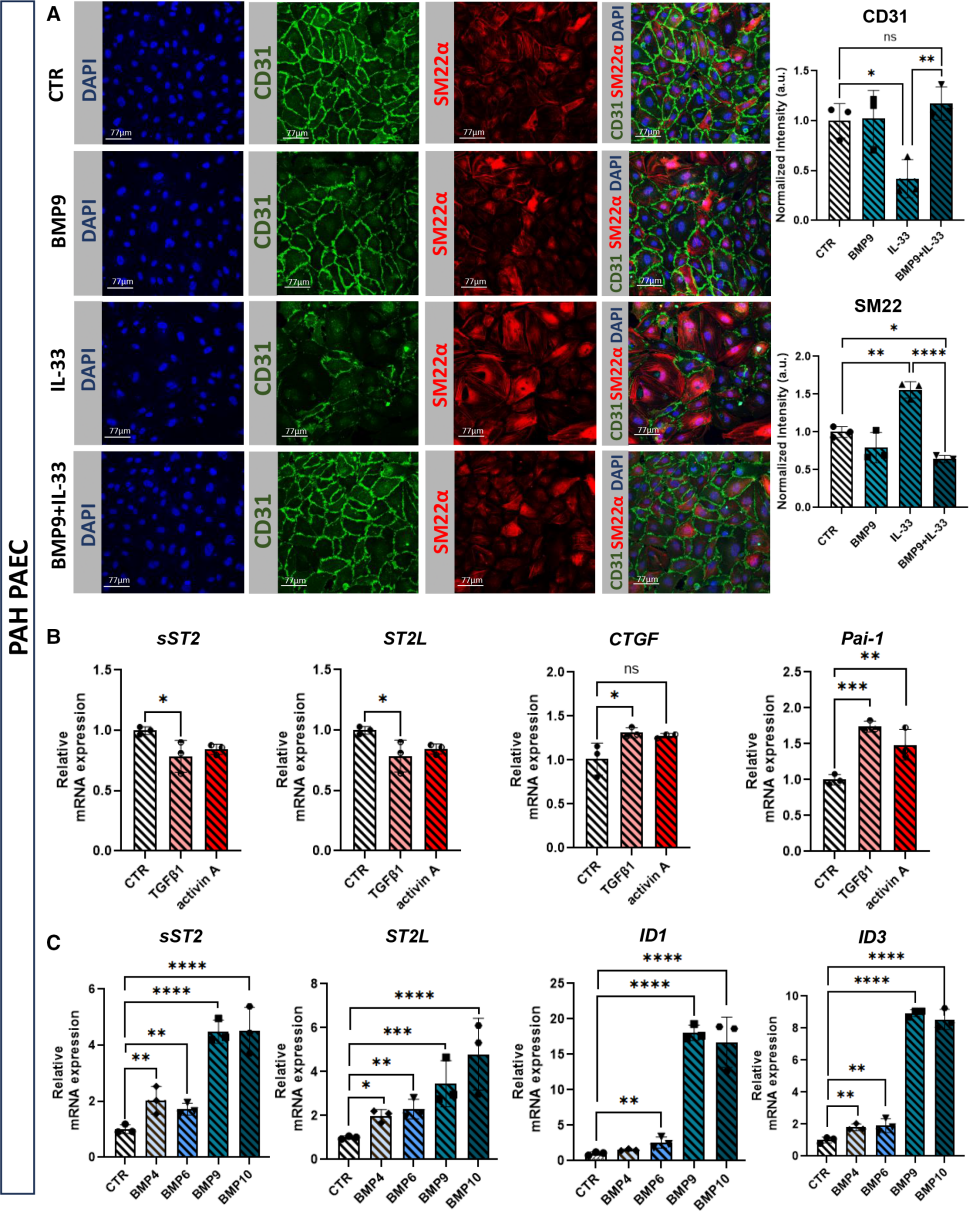
**BMP (bone morphogenetic protein) 9 protects from IL (interleukin)-33–induced endothelial-to-mesenchymal transition (EndMT) and induces sST2 (soluble supression of tumorigenicity 2) expression in pulmonary arterial endothelial cells (PAECs) from patients with pulmonary arterial hypertension (PAH) in vitro. A**, Representative immunofluorescent staining of PAH PAEC for CD31 (endothelial marker), SM22α (smooth muscle protein 22-alpha; mesenchymal marker), and 4′,6-diamidino-2-phenylindole (DAPI). Cells were treated with BMP9 (1 ng/mL), IL-33 (100 ng/mL), both, or left untreated (control [CTR]) for 3 days. Bar graphs show quantification of CD31 and SM22α intensity (technical replicates [t.n.]=3). **B**, *sST2*, *ST2L*, *CTGF*, and *Pai-1* gene expressions in PAH PAECs s after 16-hour stimulation with TGF (transforming growth factor)-β (1 ng/mL), activin A (50 ng/mL), or untreated (CTR; t.n.=3). **C**, *sST2*, *ST2L*, *ID1*, and *ID3* gene expressions in PAH PAECs after 3-hour stimulation with BMP4 (50 ng/mL), BMP6 (50 ng/mL), BMP9 (1 ng/mL), BMP10 (1 ng/mL), or untreated (CTR; t.n.=3). Statistical analysis: 1-way ANOVA with the Tukey post hoc test; *P*<0.05, *P<0.01, **P<0.001, and ***P<0.0001. Data are shown as mean±SD.

ECs from patients with PAH exhibit altered BMP9 and IL-6 responses compared with control cells.^9^ Prolonged BMP9 exposure in PAH MVECs induces EndMT-like changes, including VE-cadherin loss and increased SM22α expression. To determine whether BMP9 protects PAH PAECs from IL-33-induced EndMT, we conducted similar experiments. As in control PAECs, BMP9 alone had no effect, while IL-33 significantly reduced CD31 and increased SM22α, indicating EndMT. However, BMP9 cotreatment preserved CD31 and suppressed SM22α expression, confirming BMP9’s protective role in PAH PAECs (Figure 5A). To further assess BMP9’s role in modulating the IL-33/ST2 axis, we examined sST2 expression in PAH PAECs and MVECs. Neither TGF-β1 nor activin A induced sST2 expression in PAH PAECs (Figure 5B), control MVECs, or PAH MVECs (Figure S4A and S4C). In contrast, BMP9 and BMP10 significantly upregulated sST2 (Figure 5C; Figure S4B and S4D), highlighting their distinct role in IL-33/ST2 pathway regulation across pulmonary ECs in PAH pathogenesis.

### IL-33 Expression Is Upregulated in Pulmonary Vessels of Patients With PAH and Circulating sST2 Correlates With BMP9 Levels in Stratified PAH Groups

Since our in vitro findings suggest a crosstalk between BMP9 and sST2 in pulmonary EC implicated in the pathogenesis of PAH, we sought to investigate whether similar correlations are evident in vivo. Therefore, we measured circulating levels of sST2 and BMP9 in 79 patients with PAH. The median age of this cohort was 67 years, 53% were female patients, and 63% were classified as New York Heart Association class III or IV. Of these, 20% had pathogenic BMPR2 mutations (hereditary PAH), while the rest had idiopathic PAH. Median pulmonary artery pressure (PAP) was 48 mm Hg, and median pulmonary vascular resistance was 772 dyne/s/cm⁻⁵ (Table S5).

**Figure 6.**
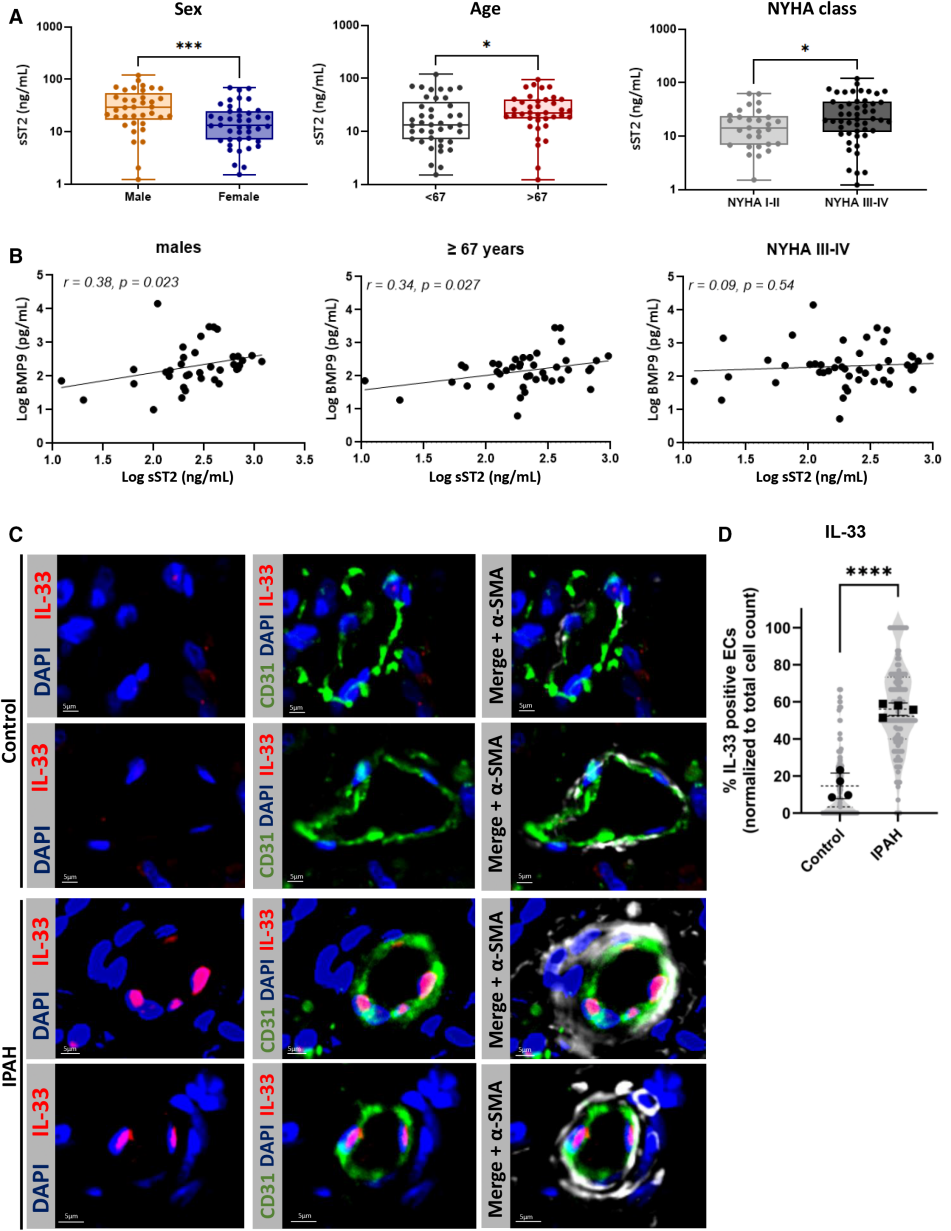
**IL (interleukin)-33 expression is upregulated in pulmonary vessels of patients with pulmonary arterial hypertension (PAH), and circulating sST2 (soluble supression of tumorigenicity 2) correlates with BMP (bone morphogenetic protein) 9 levels in stratified PAH groups. A**, Circulating levels of sST2 in patients with PAH stratified by sex, age, and New York Heart Association class (n=79). **B**, Correlation between circulating sST2 and BMP9 in substratified groups with high circulating sST2 levels. **C**, Representative images of immunohistochemical staining for IL-33, CD31, α-SMA (α-smooth muscle actin), and 4′,6-diamidino-2-phenylindole (DAPI) in lung sections from patients with PAH or healthy controls (n=4). **D**, Quantification of IL-33⁺ endothelial cells (ECs) per vessel, normalized to total ECs, from ≥30 vessels across 4 individuals per condition. Statistical analysis: (**A**) the Mann-Whitney *U* test on log-transformed values, (**B**) the Spearman correlation, and (**D**) the unpaired *t* test; *P*<0.05, *P<0.01, **P<0.001, and ***P<0.0001. Data are shown as mean±SD.

sST2 levels were significantly elevated in male patients, individuals aged >67 years, and those classified as New York Heart Association class III-IV (Figure 6A), while BMP9 levels showed no significant differences across groups (Figure S5A). Men and patients aged >67 years, circulating sST2 positively correlated with BMP9 (Figure 6B), but this association was absent in women, younger patients, and those with New York Heart Association class I-II (Figure S5B). Despite elevated sST2 levels in New York Heart Association III-IV patients, no significant correlation with BMP9 was found. Our results described above suggest that increased levels of IL-33 are associated with the pathogenesis of PAH, and its inhibition may represent a viable therapeutic strategy to mitigate endothelial remodeling in patients with PAH. To confirm increased IL-33 expression in PAH, we performed immunofluorescence on lung sections from control patients and patients with idiopahtic PAH (IPAH). α-SMA expression was elevated in the microvessels of patients with IPAH due to muscularization of the precapillary arteries, consistent with prior reports.^20^ Moreover, IL-33 expression was significantly higher in the ECs of pulmonary vessels in patients with IPAH compared with healthy controls (Figure 6C).

## Discussion

This study demonstrates that IL-33 is significantly upregulated in PAECs from patients with PAH and in pulmonary vessels of Su/Hx mice, contributing to endothelial dysfunction via EndMT. BMP9 counteracts IL-33-induced EndMT by inducing sST2, thereby neutralizing IL-33 signaling, a mechanism observed in both control and PAH-derived PAECs. We show that BMP9-mediated sST2 induction is ALK1-dependent and occurs in a dose- and time-dependent manner, requiring the presence of ALK1 receptor and sustained receptor kinase activity. Furthermore, IL-33 expression was significantly elevated in pulmonary ECs of patients with IPAH. In a cohort of patients with PAH, circulating sST2 levels were higher in men, individuals aged >67 years, and more severe PAH cases, with a positive correlation between sST2 and BMP9 levels in these stratified groups.

IL-33 has been implicated in both protective and pathological roles across cardiovascular and respiratory diseases. While it is cardioprotective in conditions like atherosclerosis, its upregulation in respiratory diseases, including chronic obstructive pulmonary disease and asthma, is linked to disease severity.^21,22^ In PAH, recent evidence suggests that prolonged IL-33 administration increases right ventricular pressure in mice, further supporting that IL-33 contributes to the progression of PAH pathogenesis.^23^ However, the exact source and regulation of IL-33 in PAH remain poorly understood. It is thought to originate from various lung cell types, including epithelial, endothelial, and possibly activated immune cells. In addition, the factors responsible for IL-33 upregulation, such as inflammation, hypoxia, or mechanical stress, require further exploration. Elevated circulating sST2 levels have been described by numerous clinical studies as a robust predictor of mortality in patients with heart failure and left ventricular systolic dysfunction.^24^ High levels of sST2 are then often associated with disease progression due to neutralization of the protective effects of IL-33 in the heart, causing maladaptive remodeling like fibrosis. In PAH, sST2 has been proposed as a prognostic biomarker with elevated levels correlating with right ventricular dysfunction and increased mortality risk.^25^ Elevated circulating sST2 levels may originate from myocardial stress, as well as from vascular remodeling in the pulmonary arteries.^26^ High sST2 levels might also reflect a compensatory mechanism to counteract the detrimental effects of elevated IL-33 in the lungs. The observed sex- and age-related differences in sST2 levels align with previous findings in cardiovascular diseases, where testosterone has been shown to increase circulating sST2.^27^ The correlation between BMP9 and sST2 in specific patient subgroups suggests a context-dependent interaction, possibly linked to advanced disease stages or systemic vascular dysfunction. However, this correlation was absent in the broader cohort, likely due to patient heterogeneity.

Mechanistically, our data reveal that BMP9 induces sST2 expression in an ALK1-dependent manner. While short-term LDN-193189 treatment failed to block *sST2* induction at 3 hours, siRNA-mediated *ALK1* knockdown abolished the response, demonstrating that ALK1 is essential for transcriptional activation. BMP9 triggered sST2 expression in a dose-dependent manner beginning at 0.1 ng/mL and is consistent with its high-affinity interaction with ALK1, while BMP6, primarily signaling via ALK2, had no effect. Together, these findings indicate that BMP9 induces sST2 expression through ALK1 and suggest that canonical ALK1 signaling may activate a downstream regulatory mechanism that sustains or stabilizes sST2 expression beyond the initial transcriptional trigger. The partial insensitivity to LDN-193189 at early time points likely reflects context-specific limitations in blocking ALK1 activity in PAECs, rather than true receptor independence.

Despite these important insights, our study has some limitations. The lack of in vivo validation for BMP9-IL-33-sST2 interactions restricts our mechanistic understanding. IL-33 was undetectable in patient plasma, suggesting local sequestration or rapid degradation. Distinguishing between endogenous sST2 and the transmembrane isoform ST2L at the protein level remains technically challenging, and addressing this gap would enhance elucidation of their distinct roles of sST2 and ST2L in cellular and physiological contexts in PAH.

Additional limitations may include the following: EndMT has been demonstrated to be a hallmark of endothelial dysfunction in PAH although its specific contribution to disease development remains unclear. Previous studies have proposed that preventing or reversing EndMT typically reduces medial thickening and muscularization, restores endothelial markers (eg, CD31 and VE-cadherin), attenuates mesenchymal/fibrotic markers (eg, α-SMA, vimentin, and collagen I/III), lowers pulmonary pressures, and ameliorates right ventricular hypertrophy.^28–30^ However, to the best of our knowledge, no selective EndMT inhibitor has been identified, and researchers rely on pleiotropic inhibitors of TGF-β or Wingless-Related Integration Site (Wnt) signaling, anti-inflammatory compounds, or epigenetic modulators. In our study, we did not directly test the downstream consequences of EndMT inhibition on pulmonary vascular remodeling or right ventricular function in vivo. Second, our patient-derived PAECs were obtained at advanced disease stages and exposed to combination therapies, which may bias effect sizes (eg, blunting or exaggerating responsiveness to BMP9 or IL-33), whereas blood samples were collected at diagnosis, which may influence interpretations. Third, EndMT is heterogeneous across vascular beds. Limited primary material prevented systematic testing in all endothelial subtypes (eg, PAH MVECs), which may introduce imprecision when extrapolating our findings to the entire pulmonary microvasculature.

It is important to acknowledge that the IL-33/ST2 signaling axis exhibits considerable biological complexity that may challenge in vitro investigation. IL-33 functions both as a nuclear chromatin-associated factor and a secreted cytokine, with extracellular activity highly context-dependent and influenced by proteolytic processing, oxidative environment, and cellular origin.^31^ sST2 is widely recognized as a soluble decoy receptor neutralizing IL-33 by preventing its interaction with the membrane-bound ST2L, but recent hypotheses suggest that sST2-IL-33 complexes might stabilize IL-33 by protecting it from oxidation or proteolytic cleavage, potentially extending its half-life.^32^ However, current evidence does not convincingly demonstrate a functional role for these complexes beyond IL-33 sequestration. Moreover, the low abundance, rapid oxidation, and instability of circulating IL-33 pose technical challenges for its reliable detection and quantification, further limiting mechanistic insight. Our findings are interpreted within the framework of sST2 acting as an inhibitor of IL-33 signaling, but alternative or noncanonical mechanisms may contribute to BMP9’s modulation of IL-33-induced EndMT, and merit further investigation.

Therapeutically, BMP9 has been explored in PAH due to its ability to enhance BMPR2 signaling.^17^ However, its effects are highly context-dependent, with evidence for both protective and pathological roles, including EndMT induction in PAH MVECs. The complexity of BMP9 signaling, including its context-dependent effects on endothelial function and potential to induce EndMT, raises concerns about its therapeutic application in PAH. Agonistic strategies that enhance BMP9 activity may, at higher doses or prolonged exposure, signal via lower-affinity receptors, potentially promoting inflammation or tissue mineralization.^33^ In PAH endothelium, chronic BMP9 exposure can prime or exacerbate EndMT, particularly in a proinflammatory milieu, indicating that context and dosing are critical.^9^ Conversely, BMP9/10 blockade (eg, ALK1-extracellular domain–like approaches) risks hereditary hemmorrhagic talangiectasia (HHT)-like vascular phenotypes, systemic vasodilation, arteriovenous shunting, pulmonary vascular dilatation, altered vasodilator/vasoconstrictor balance, and cardiac remodeling, highlighting cardiovascular safety concerns.^33,34^ Given these complexities, alternative approaches that modulate vascular remodeling without BMP9-associated risks are of great interest. One such approach is sotatercept, an ACVRIIA-Fc ligand trap against activins and GDFs (Growth Differentiation Factors) that restores the balance between the proproliferative activin pathway and the antiproliferative BMP pathway, improving exercise capacity and reducing clinical worsening.^35^ IL-33 inhibition may offer a more targeted approach to prevent endothelial dysfunction without the complexities associated with BMP9 signaling. Monoclonal antibodies targeting IL-33, such as tozorakimab and itepekimab, are currently in clinical trials for chronic obstructive pulmonary disease (NCT05166889 and NCT05158387), asthma (NCT04570657), and acute respiratory failure (NCT05624450) and might be repurposed for PAH. However, systemic inhibition of the IL-33/ST2 signaling axis presents some safety concerns due to its pleiotropic roles. IL-33/ST2L acts as a mechanosensitive signal to physiologically limit adverse cardiac remodeling. Its loss compromises myocardial integrity, increasing susceptibility to hypertrophy and fibrosis after pressure overload and worsening outcomes following myocardial infarction by removing its inhibitory effect on cardiomyocyte apoptosis and its support for recovery.^32,36,37^ Beyond the heart, systemic blockade may disrupt immune regulation, as IL-33 promotes the function and clonal expansion of regulatory T cells; impaired signaling has been clinically linked to conditions such as inflammatory bowel disease. Furthermore, IL-33 is an alarmin released by barrier tissues in response to helminths to coordinate early type 2 immune defense and is integral to pulmonary tissue repair after viral challenge.^38^ While localized IL-33 suppression to limit EndMT in PAH is a promising strategy, it must be carefully balanced against the high risk of losing beneficial IL-33 effects in the heart and at barrier surfaces. Consequently, developing anti-IL-33 and anti-ST2 agents requires precise optimization of drug timing, delivery method, and rigorous patient stratification.

Therapeutic modulation of sST2 or ST2L could refine IL-33 activity though no selective blocking agents currently exist for these isoforms. In addition to this limitation, the restricted availability of primary patient-derived materials compromised our ability to perform experiments across all EC types, including MVECs from patients with PAH. However, the observed induction of sST2 by BMP9 in different ECs suggests that our findings may be broadly applicable.

In summary, our findings offer an alternative yet complementary perspective, highlighting the potential of targeting IL-33 signaling as a strategy to mitigate endothelial dysfunction in PAH. Unlike BMP9-based therapies, which have shown both protective and pathological effects depending on the context, IL-33 inhibition could offer a more targeted strategy to prevent EndMT and vascular remodeling. By focusing on IL-33 inhibition, this approach may complement existing strategies while addressing some of the challenges posed by the dual roles of BMP9 in PAH pathogenesis.

## Perspectives

This study reveals a previously underappreciated role for IL-33 as a driver of EndMT in PAH, marked by elevated IL-33 expression in both patient-derived ECs and the Su/Hx mouse model. We identified a protective mechanism, whereby BMP9 upregulates the decoy receptor sST2, thereby antagonizing IL-33 signaling and mitigating endothelial dysfunction. This BMP9-sST2 axis offers new insight into how vascular homeostasis is preserved in PAH and advances our understanding of cytokine-mediated vascular remodeling.

Therapeutically, our findings position IL-33 inhibition as a promising strategy to counteract EndMT and pathological vascular remodeling in PAH. Monoclonal antibodies targeting IL-33, currently in clinical trials for other inflammatory lung diseases, could potentially be repurposed for PAH, offering a more selective approach that may circumvent the broader and context-dependent action of BMP9-based therapies.

Future research should focus on validating IL-33 inhibition in in vivo models of PAH and investigate the broader therapeutic potential of modulating sST2 expression across diverse vascular beds. Elucidating the upstream regulators of sST2 expression and molecular determinants of IL-33 responsiveness in ECs will be important to further refine these strategies. Ultimately, by uncovering this signaling axis, our study paves the way for more targeted and mechanistically informed interventions aimed at halting or reversing vascular remodeling in PAH.

## ARTICLE INFORMATION

### Acknowledgments

Schematic figures were created with biorender.com (licensed to G. Sanchez-Duffhues).

### Author Contributions

C. Becher conceptualized and designed the experiments. C. Becher and E.J. Groeneveld performed all experiments. C. Becher analyzed the data. R. Quarck provided human plasma and analyzed the clinical data. B. Neep and X. Pan isolated and provided primary cells. H.J. Bogaard, P.B. Yu, and F. de Man provided human and mouse materials. G. Sanchez-Duffhues supervised the project. C. Becher designed the figures and wrote the manuscript. All authors revised and edited the manuscript. G. Sanchez-Duffhues and M.J. Goumans provided resources and funding acquisition.

### Sources of Funding

This work was supported by the FWO Scientific Research Network (grant W0014200N). C. Becher and M.J. Goumans are sponsored by the Netherlands Cardiovascular Research Initiative (the Dutch Heart Foundation, the Dutch Federation of University Medical Centers, the Netherlands Organization for Health Research and Development, and the Royal Netherlands Academy of Arts and Sciences), PHAEDRA-IMPACT (Dutch Cardiovascular Alliance, DCVA), and DOLPHIN-GENESIS (CardioVasculair Onderzoek Nederland, CVON). C. Becher is sponsored by the European Joint Program on Rare Diseases and the Company of Biologists. M.J. Goumans is supported by Regenerative Medicine Crossing Borders. G. Sanchez-Duffhues is supported by the grants Ramón y Cajal RYC2021-030866-I, PID2022-141212OA-I00, and CNS2023-145432 from the Spanish Ministry of Science and Innovation, the British Heart Foundation (BHF)-German Centre for Cardiovascular Research (DZHK)-Dutch Heart Foundation (DHF), 2022/23 award PROMETHEUS (grant 02-001-2022-0123), and the Foundation Eugenio Rodriguez Pascual (grant FERP-2023-058). The authors are grateful to the Belgian patients with pulmonary hypertension.

### Disclosures

None.

### Supplemental Material

Supplemental Materials and Methods

Tables S1–S5

Figures S1–S6

Unedited Blots

## Supplementary Material


