# Supplementary material for: BMP9 Modulates IL-33 Signaling to Mitigate EndMT in Pulmonary Arterial Hypertension
Source: Hypertension. 2026 Jan 22;83(2):e24916. doi: 10.1161/HYPERTENSIONAHA.125.24916 (PMC12822782; doi:10.1161/HYPERTENSIONAHA.125.24916)
Supplement: Supplementary file 1 [file hyp-83-e24916-s001.docx]

Supplemental Material

**BMP9 Modulates IL-33 Signaling to Prevent EndMT in Pulmonary Arterial Hypertension**

Short title: Regulation of EndMT in PAH by BMP9 and IL-33

Clarissa Becher^1^, Esmee J Groeneveld^1^, Rozenn Quarck^2^, Beau Neep^3,4^, Pan Xiaoke^3,4^, Robert Szulcek^1,3,5,^, Ly Tu^6^, Christophe Guignabert^6^, Harm Jan Bogaard^3,4^, Paul B Yu^7^, Frances de Man^3,4^, Gonzalo Sanchez-Duffhues^1,8⊥^, Marie-José Goumans^1⊥^

^⊥^These authors contributed equally.

^1^Department of Cell and Chemical Biology, Leiden University Medical Center, Einthovenweg 20, 2333 ZC, Leiden, the Netherlands.

^2^Laboratory of Respiratory Diseases and Thoracic Surgery, Department of Chronic Diseases and Metabolism, KU Leuven-University of Leuven, Belgium

^3^ PHEniX laboratory, Department of Pulmonary Medicine, Amsterdam University Medical Center (UMC) (Vrije Universiteit), 1081 HV Amsterdam, The Netherlands.

^4^Amsterdam Cardiovascular Sciences, Pulmonary Hypertension & Thrombosis, Amsterdam, The Netherlands

^5^Laboratory of *in vitro* Modeling Systems of Pulmonary and Thrombotic Diseases, Institute of Physiology, Charité - Universitätsmedizin Berlin, Berlin, Germany.

^6^Université Paris-Saclay, Hypertension Pulmonaire: Physiopathology and Innovation Thérapeutique, HPPIT, Faculté de Médecine, Le Kremlin-Bicêtre, France.

^7^Division of Cardiovascular Medicine, Department of Medicine, Brigham and Women's Hospital, Harvard Medical School, Boston, MA, 02115, USA

^8^Nanomaterials and Nanotechnology Research Center (CINN-CSIC), Health Research Institute of Asturias (ISPA), 33011 Oviedo, Asturias, Spain

Address for correspondence: Gonzalo Sanchez-Duffhues, Nanomaterials and Nanotechnology Research Center (CINN-CSIC), Health Research Institute of Asturias (ISPA), 33011 Oviedo, Asturias, Spain. Email: [g.s.duffhues@cinn.es](mailto:g.s.duffhues@cinn.es) and Marie-José Goumans, Department of Cell and Chemical Biology, Leiden University Medical Center, Einthovenweg 20, 2333 ZC Leiden, the Netherlands. Tel: 31-71 526 9264; Fax: 31-071 526 8270; E-mail: M.J.T.H.Goumans@lumc.nl

Expanded Materials and Methods

1. EndMT assay (immunofluorescent staining)

Cells were washed in cold PBS, fixed in 4% paraformaldehyde (20 min, RT), quenched with 2 mg/mL glycine, and permeabilized with 0.2% Triton-X (10 min). Blocking was performed with 5% BSA (Sigma-Aldrich, #A-6003), followed by overnight incubation (4°C) with primary antibodies against CD31 (R&D Systems, #AF3628), VE-Cadherin (Cell Signaling, #2158), and SM22α (Abcam, #ab14106) in 1% BSA/PBS (working concentrations are stated in supplementary table S3). After washing with 0.5% BSA/0.05% Tween-20 in PBS, samples were incubated at RT (1h, dark) with Alexa-conjugated secondary antibodies. Samples were preserved in ProLong Gold with DAPI (Thermo Fisher) and imaged using a confocal microscope (SP8, Leica Microsystems). ImageJ was used for quantification by measuring VE-cadherin or CD31 and SM22α intensity in ten individual cells from three random locations per well, normalized to unstimulated controls.

1. Western Blotting

Lysates were prepared in RIPA buffer with protease (Roche, #11836145001) and phosphatase inhibitors (10 mM sodium fluoride, Sigma, #7681-49-4; 400 μM sodium orthovanadate, Sigma, #S6508). Protein concentrations were determined using the Pierce BCA Assay Kit (Thermo Fisher, #23235). Equal protein amounts (20 μg) were mixed with Laemmli buffer, resolved via SDS-PAGE (10% polyacrylamide gels), and transferred onto methanol-activated 45 μm PVDF membranes (Merck Millipore, #IPVH00010). Membranes were blocked (10% non-fat dry milk in TBST, 1h, RT) and incubated overnight (4°C) with primary antibodies in TBST: phospho-p38 MAPK (Thr180/Tyr182) (Cell Signaling, #4631), phospho-IκB-α (Ser32/36) (Santa Cruz, #sc-101713), and vinculin (Sigma-Aldrich, #V9131) as a loading control (Table S2). After TBST washes, membranes were incubated (1h, RT) with secondary antibodies (1:10,000) anti-mouse HRP (Promega, #W4021) or anti-rabbit HRP (Invitrogen, #31458) in 10% milk/TBST. Detection was performed using ECL (Western Bright Quantum HRP, Advansta, #K-12042-D20), and signals were visualized on a ChemiDoc Imaging System (Bio-Rad).

1. Immunohistochemical staining

Lung sections were deparaffinized, hydrated, and antigen retrieval was performed by boiling in Antigen Retrieval Buffer (10 mM Tris, pH 9, 1 mM EDTA, 0.05% Tween-20) for 20 min in a pressure cooker. After blocking (1% BSA in 0.1% Tween-PBS), sections were incubated overnight (4°C) with primary antibodies against PECAM-1 (R&D Systems, #AF3628), αSMA (Sigma, #A2547), and IL-33 (R&D Systems, #AF3626-SP) (Table S2). The next day, sections were incubated with Alexa-conjugated secondary antibodies, washed with 0.1% Tween-20/PBS, stained with DAPI (Thermo Scientific, #62248), and mounted with ProLong Gold Antifade (Invitrogen, #P26930). Slides were scanned using the Panoramic 250 slide scanner (3DHISTECH, v1.23) and analyzed in Case Viewer (3DHISTECH, v2.3). IL-33⁺ endothelial cells (EC) were quantified using ImageJ and normalized to total EC nuclei per vessel.

**Supplementary Tables**

Supplemental table S1 | Growth factors and cytokines

| Growth factors | vendor | Catalogue # |
| --- | --- | --- |
| BMP4 | R&D systems | 314-BP-010/CF |
| BMP6 | R&D R&D systems | 507-BP-020/CF |
| BMP9 | R&D R&D systems | 3209-BP-010/CF |
| BMP10 | R&D R&D systems | 2926-BP-025/CF |
| Activin A | R&D R&D systems | 338-AC-010/CF |
| TGF-β | R&D R&D systems | 240-B-010/CF |
| IL-33 | Prospec | CYT-425 |
| Recombinant ST2 | R&D R&D systems | 523-ST-100 |
| LDN-193189 | Selleckchem | S2618 |

Supplemental table S2 | Characteristics of PAH patients and control cells

|  | Control | PAH |
| --- | --- | --- |
| Participant (n) | 5 | 7 |
| Female (%) | 40 | 100 |
| Age | 72 [69-74] | 40 [31-43] |
| IPAH (%) | NA | 57 |
| HPAH (%) | NA | 43 |
| mPAP | NA | 61.5 [51-76.5] |
| NT-proBNP | NA | 1806 [1375-4020] |

Supplemental table S3 | Antibodies

| Target antigen | vendor | Catalogue # | Working concentration |
| --- | --- | --- | --- |
| Transgelin (SM22α) | Abcam | ab14106 | 1:250 |
| Pecam (CD31) | R&D systems | AF3628 | 1:100 (IF), 1:200 (IHC, human) |
| Ve-Cadherin | Abcam | Ab205336 | 1:150 (IHC,mouse) |
| IL-33 | R&D systems  Proteintech | AF3626  12372-1-AP | 1:100  1:800 |
| Alpha-SMA | Sigma | A2547 | 1:40,000 |
| Alexa Fluor™ Plus 647 Phalloidin | Invitrogen | A30107 | 1:200 |
| Phosphor-p38 MAPK (Thr180/Tyr182) | Cell Singling | 4631 | 1:1000 |
| phospho-IκB-α (Ser32/36) | Santa Cruz Biotech. | Sc-101713 | 1:1000 |
| Phospho-SMAD1(Ser463/465) /SMAD5(Ser463/465)/ SMAD9 (Ser465/467) | Cell Signaling | 13820 | 1:1000 |
| SMAD1 | Cell Singling | 6944 | 1:1000 |
| Vinculin | Sigma-Aldrich | V9131 | 1:1000 |
| Human ST2L/IL-33R | R&D systems | MAB523 | 1ug/mL |
| Mouse IgG Isotype Control | R&D systems | MAB002 | 1ug/mL |
| Alexa Fluor™ 488 | Thermo Fisher | A11055 or A21206 | 1:250 |
| Alexa Fluor™ 555 | Thermo Fisher | A31572 | 1:250 |
| Alexa Fluor™ 647 | Thermo Fisher | A21447 | 1:250 |

Supplemental table S4 | Primer sequences for qPCR

| **Gene** | **Forward 5’🡪 3’** | **Reverse 5’🡪 3’** | **bp** | **Annealing Temp (C°)** | **Gene Bank No.** | **Specificity/Notes** |
| --- | --- | --- | --- | --- | --- | --- |
| *ID1* | CTGCTCTACGACATGAACGG | GAAGGTCCCTGATGTAGTCGAT | 124 | 60 | NM_181353.3, NM_002165.4 | No additional specific hits in RefSeq mRNA. Partial mismatch binding observed for ID2 (NM_002166.5). |
| *ID3* | CACCTCCAGAACGCAGGTGCTG | AGGGCGAAGTTGGGGCCCAT | 99 | 60 | NM_002167.5 | Only ID3 mRNA amplified; no off-target products detected in RefSeq mRNA |
| *SMAD6* | ACAAGCCACTGGATCTGTCC | ACATGCTGGCGTCTGAGAA | 102 | 60 | NM_005585.5 | Intended product on SMAD6; matches also to predicted SMAD6 XM variants; no other gene targets. |
| *Serpine1 (Pai-1)* | CACAAATCAGACGGCAGCACT | CATCGGGCGTGGTGAACTC | 85 | 60 | NM_000602.5; NM_001386460.1; NM_001386458.1; NM_001386463.1; NM_001386464.1; NM_001386461.1; NM_001386465.1; NM_001386466.1 | Amplifies SERPINE1 across listed transcript variants; no non-SERPINE1 amplicons detected in RefSeq mRNA. |
| *CCN2 (CTGF)* | TTGCGAAGCTGACCTGGAAGAGAA | AGCTCGGTATGTCTTCATGCTGGT | 121 | 60 | NM_001901.4 | Single intended product on CCN2; no off-target amplicons. |
| *sST2* | CTCCAAGTTCATCCCCTCTG | GATCCAAAACCCCATTCTGTT | 197 | 55 | NM_003856.4 | Forward primer in unique **exon 1** (sST2-specific)  Intended amplicon on IL1RL1 **sST2** transcript; no product on ST2L |
| *ST2L* | GCACTTTGTTCACCAGATTCT | CCAGGTAGCATATCTCTCCCA | 87 | 55 | NM_016232.5 | Primers in regions **absent in sST2**  Intended product on IL1RL1 **ST2L** transcript; no product on sST2 |
| *IL-33* | GGAGTGCTTTGCCTTTGGTA | TCATTTGAGGGGTGTTGAGA | 243 | 55 | NM_033439.4  NM_001314044.2  NM_001314045.2 NM_001353802.2 | Produces amplicons on multiple IL33 transcript variants only; no non-IL33 amplicons. |
| *MyD88* | AAAGAGGTTGGCTAGAAGGC | CAAGGCGAGTCCAGAACCA | 254 | 55 | NM_001172567.2 | \| Single intended product on MYD88;  no off-target amplicons. \| \| --- \|  \|  \| \| --- \| |
| *IL1RAP* | CACTTCTGTGGTGTGTAGTGA | AATGCAACTTTGCTG CAATAT | 376 | 55 | NM_002182.4; NM_134470.4; NM_001167928.2; NM_001167929.2; NM_001167930.2; NM_001167931.2; NM_001364879.1; NM_001364880.2; NM_001364881.2 | Intended product across multiple IL1RAP transcript variants; no non-IL1RAP amplicons. |
| *IL-8* | CTGTTAAATCTGGCAACCCTAGTCT | CAAGGCACAGTGGAACAAGGA | 376 | 60 | NM_000584.4; NM_001354840.3 | Intended product on CXCL8 transcript variants; no non-CXCL8 amplicons. |
| *GAPDH* | AGCCACATCGCTCAGACAC | GCCCAATACGACCAAATCC | 66 | 60 | NM_002046.7; NM_001289746.2; NM_001357943.2; NM_001289745.3 | Housekeeping  Intended product across GAPDH transcript variants; no non-GAPDH amplicons. |
| *ARP (RPLP0)* | CACCATTGAAATCCTGAGTGATGT | TGACCAGCCGAAAGGAGAAG | 116 | 60 | NM_001002.4; NM_053275.4 | Housekeeping  Intended product on RPLP0 (ARP) variants. Primer-BLAST lists isolated **reverse-primer** alignments to unrelated transcripts (e.g., CRB1/ITSN1) but **no paired-primer amplicons**—thus no off-target PCR products. |

Supplemental table S5 | Participants baseline characteristics

| **Participants (n)** | **N=79** |
| --- | --- |
| **Female, %** | 53 |
| **Age (years)** | 67 [61-69] |
| **Idiopathic PAH (%)** | 80 |
| **Heritable PAH (%)** | 20 |
| **Serum NT-proBNP, ng.L^-1^** | 1377 [907-1810] |
| **BMI (kg.m^-2^)** | 25.6 [24.9-26.5] |
| **mPAP, mmHg** | 48 [45-51] |
| **PVR, dyne.s.cm^-5^** | 772 [642-856] |
| **Cardiac Index, L.min.m^2^** | 2.08 [1.94-2.30] |
| **RAP, mmHg** | 7 [6-8] |
| **6MWD, m** | 300 [260-357] |
| **NYHA FC, %**  **I**  **II**  **III**  **IV** | 5  31  49  14 |
| **BM9, pg.mL-1** | 1377 [907-1810] |
| **sST2, ng/mL -1** | 19.6 [14.5-24.3] |

**Baseline characteristics.** BMI, body mass index; mPAP, mean pulmonary arterial pressure; PVR, pulmonary vascular resistance; RAP, right atrial pressure; NYHA FC, New York Heart Association functional class; 6MWD, 6-minute walking distance. Results are expressed as median [95% confidence interval]

Supplementary Figures

**
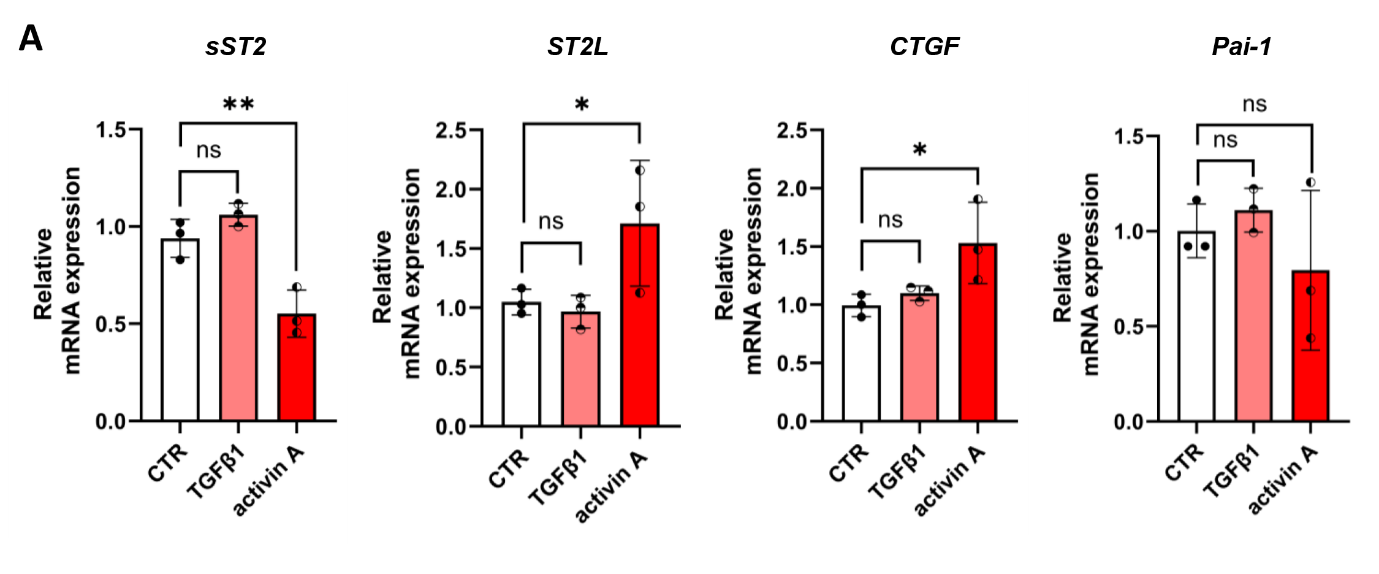
**

**Figure S1: TGF-β ligands do not change sST2 expression in PAECs *in vitro.*** (A) Gene expression analysis of *sST2, ST2L, CTGF* and *Pai-1* in control PACEs stimulated for 3h with TGF-β (1ng/mL), ActivinA (50ng/mL) or left untreated (CTR).

**
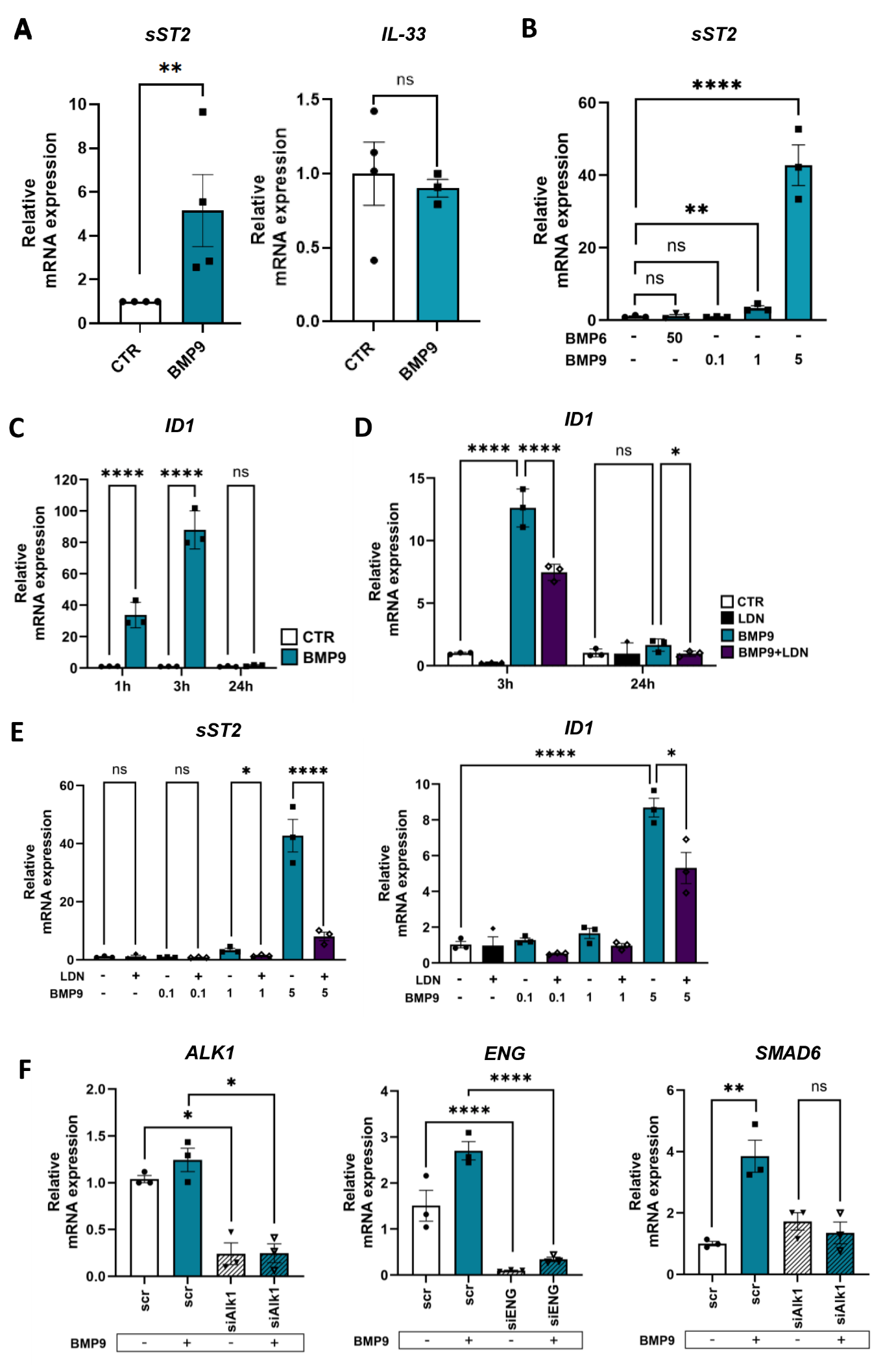
**

**Figure S2: BMP9 induces sST2 expression in a dose- and time-dependent manner through ALK1 signaling in PAECs *in vitro*.** (A) mRNA expression of sST2 and IL-33 in PAEC after 3-hour BMP9 (1 ng/mL) stimulation compared to unstimulated controls. Each data point represents three biological replicates per donor (B) Dose-dependent increase in sST2 mRNA following 24-hour stimulation with 0.1, 1, or 5 ng/mL BMP9. (C) mRNA expression of ID after 1, 3, or 24 hours of BMP9 stimulation (1 ng/mL). (D) ID1 mRNA expression in PAECs pre-treated with LDN-193189 (120 nM, 30 min) followed by BMP9 (1 ng/mL) stimulation for 3 or 24 hours. (E) mRNA expression of sST2 and ID1 following 24-hour stimulation with increasing concentrations of BMP9 in the presence or absence of LDN-193189 (120 nM). (F) Alk1, ENG and SMAD6 mRNA expression in PAECs transfected with siRNA targeting ALK1 or ENG and stimulated with BMP9 (1 ng/mL, 3h). Statistical analysis: (A) unpaired Student’s t-test; (B, E-F) one-way ANOVA with Tukey’s post-hoc test for multiple comparisons; (C-D) two-way ANOVA with Tukey’s post-hoc testing for multiple comparisons. *p<0.05, **p<0.01, ***p<0.001, ****p<0.0001, ns = not significant. Data shown as (A) mean ± SEM, (B-F) mean ± SD.


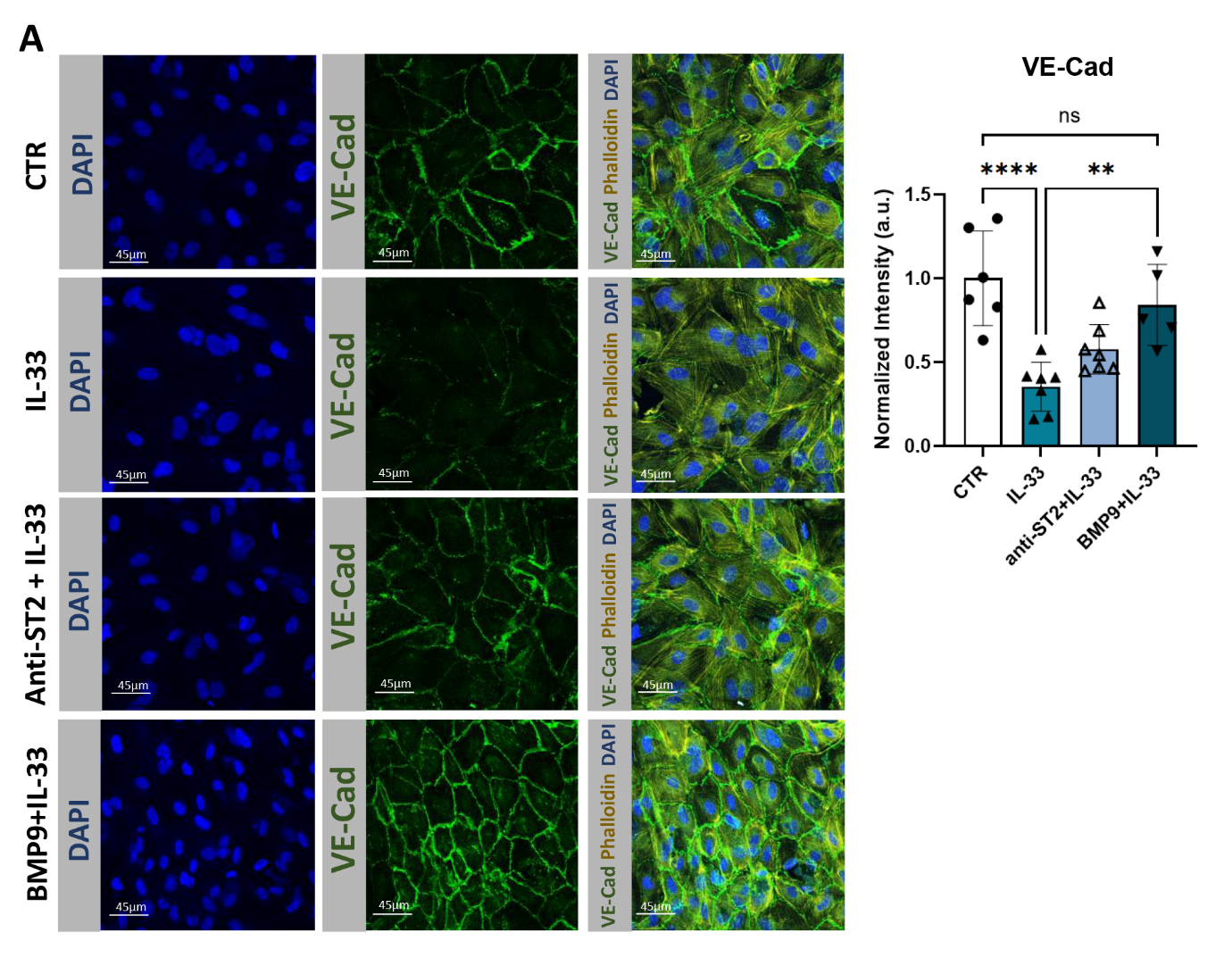


**Figure S3: BMP9 rescues IL-33-induced EndMT equally effective anti-ST2L antibody in PAECs *in vitro*.** (A) Representative immunofluorescent staining of PAEC for the endothelial marker Ve-Cad, phalloidin, and DAPI. Cells were either left unstimulated, or were pre- treated with BMP9 (1ng/mL) for 3h or anti-ST2L antibody (1ug/mL) for 30 min, followed by stimulation with IL-33 (100ng/mL) for 3 days. Bar graphs represent quantifications of VE-Cad intensity.


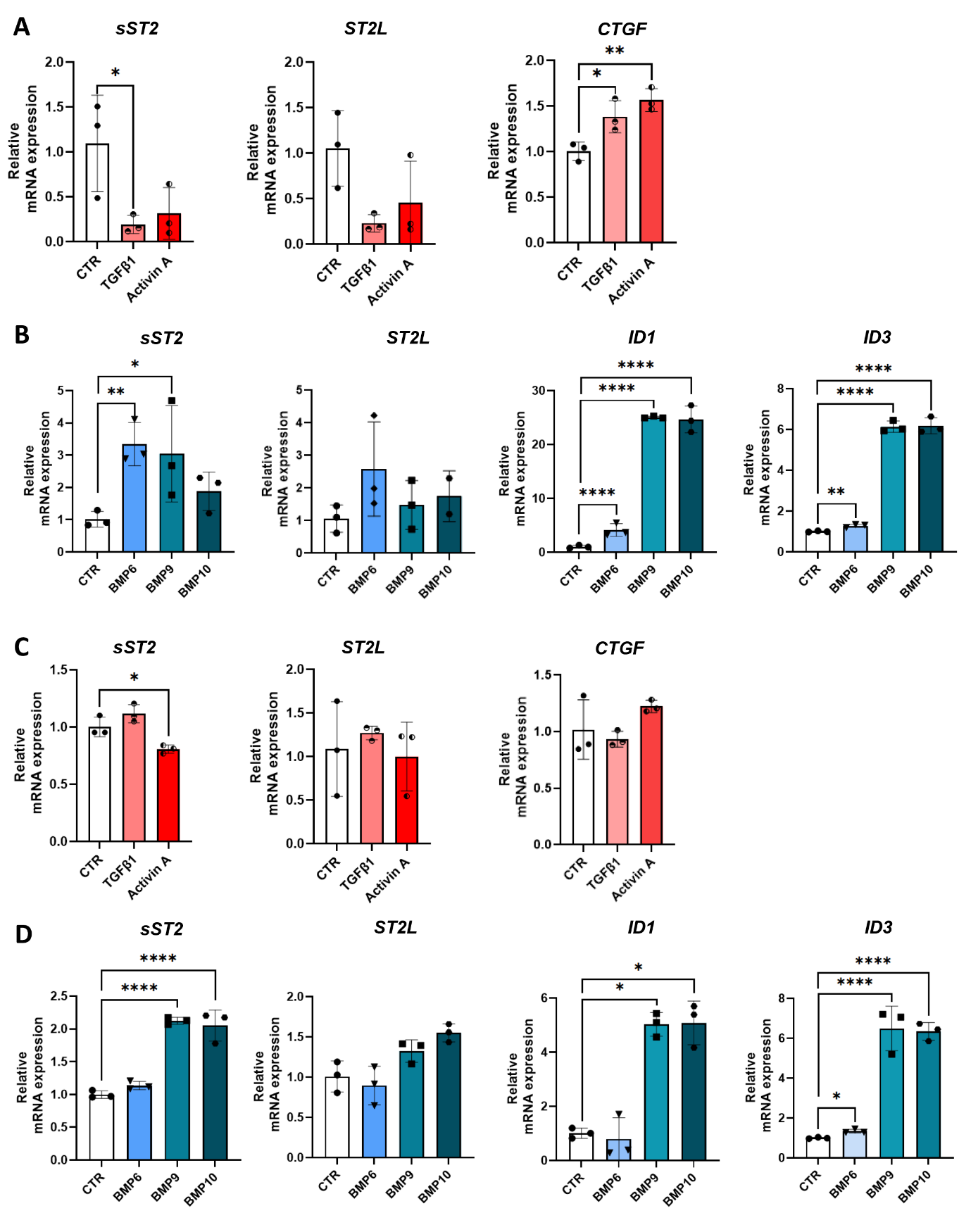


**Figure S4: BMP9 induces sST2 expression *in vitro* in control MVECs and MVECs from PAH patients.** (A) Gene expression analysis of *sST2, ST2L, CTGF* and *Pai-1* in control MVECs or (C) PAH MVECs stimulated for 16h with TGF-β (1ng/mL), ActivinA (50ng/mL) or left untreated (CTR). (B) Gene expression analysis of *sST2, ST2L, ID1* and *ID3* in control MVECs or (D) PAH MVECs stimulated for 3h with BMP6 (50ng/mL), BMP9 (1ng/mL), BMP10 (1ng/mL), or left untreated (CTR)(N=3). Statistical differences were tested using one-way ANOVA; *p<0.05, **p<0.01, ***p<0.001, ****p<0.0001. Graphs are displayed as mean + SD.

**
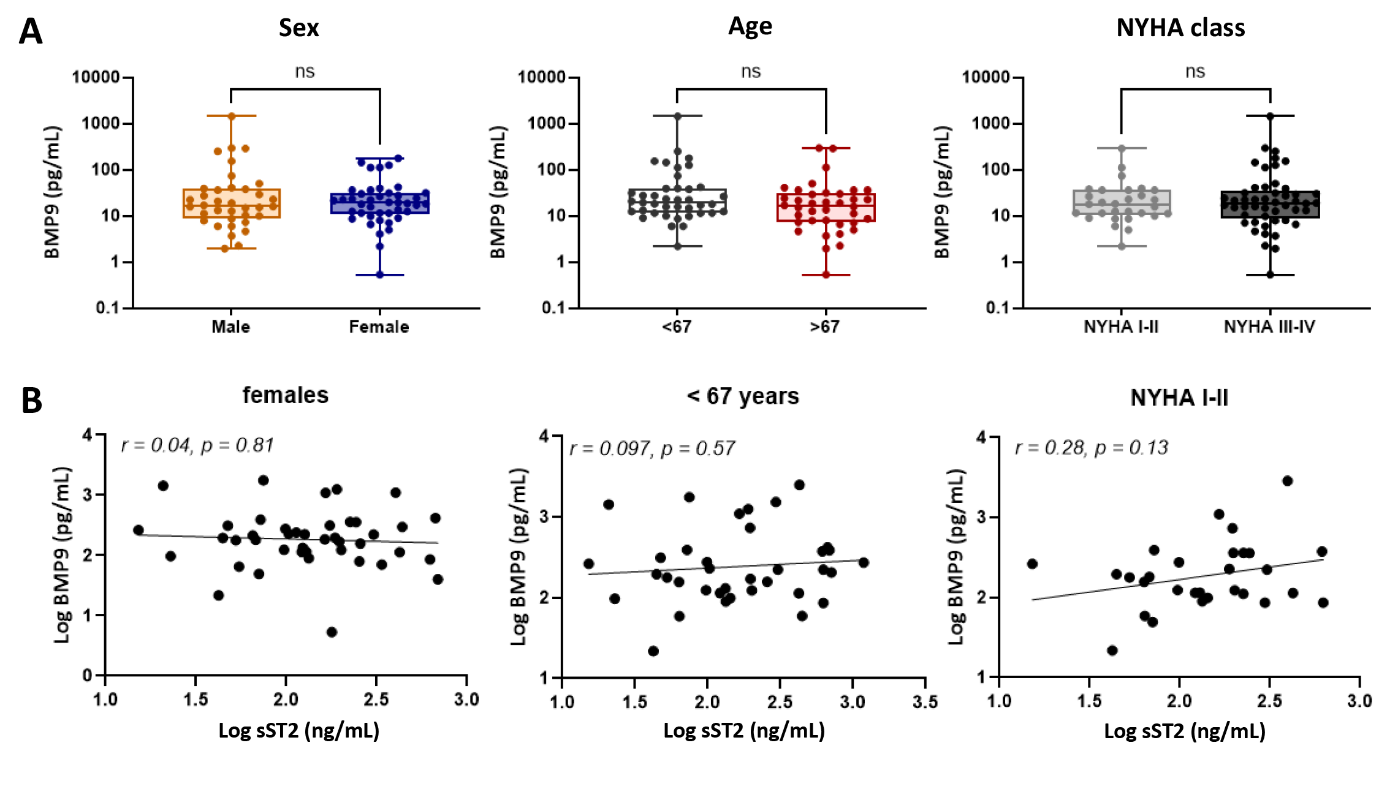
Figure S5. No positive correlation between sST2 and BMP9 levels in stratified PAH Groups.** (A) Circulating levels of BMP9 in PAH patients stratified by sex, age and NYHA class (N=79). (B) Correlation between circulating sST2 and BMP9 in sub-stratified groups with low circulating sST2 levels.

Supplemental Staining’s

**
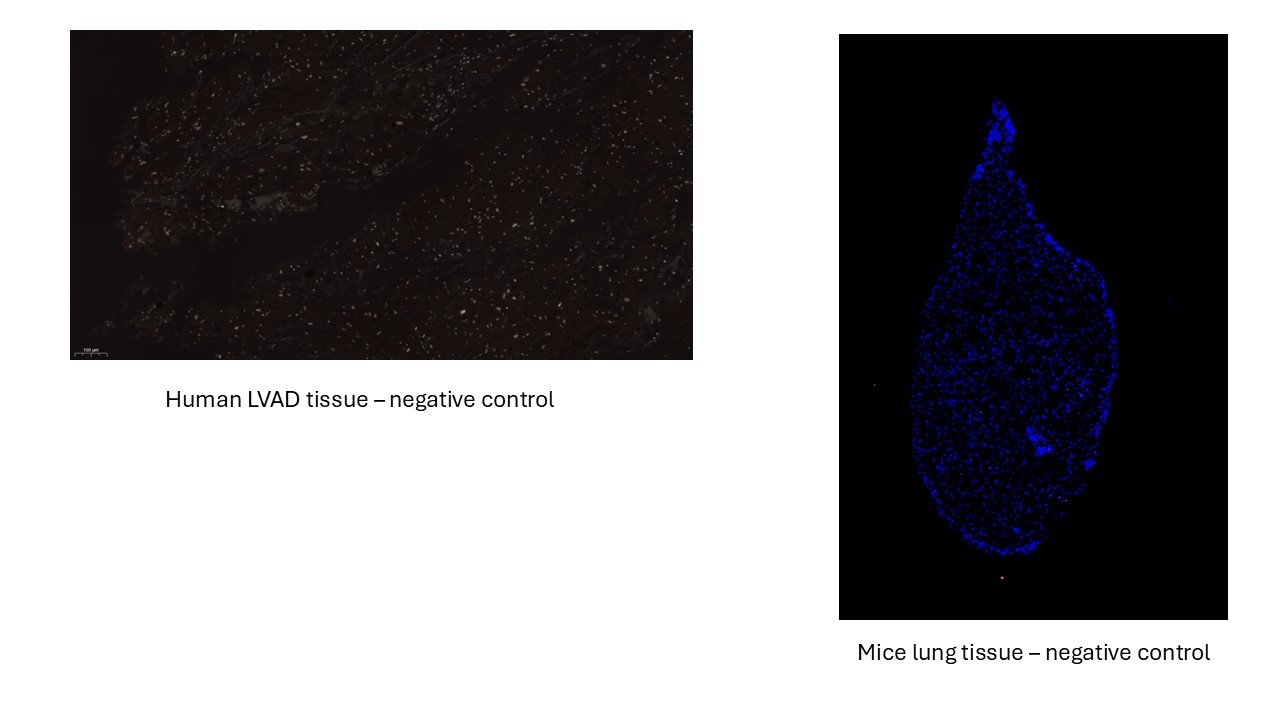
**

**Figure S6**. Representative immunohistochemical images of negative control staining’s for (left) human IL-33 antibody on human tissue on a left ventricular assist device (LVAD) and (right) for mouse IL-33 antibody on a mouse lung tissue slide
