# Supplementary material for: BMP9 Modulates IL-33 Signaling to Mitigate EndMT in Pulmonary Arterial Hypertension
Source: Hypertension. 2026 Jan 22;83(2):e24916. doi: 10.1161/HYPERTENSIONAHA.125.24916 (PMC12822782; doi:10.1161/HYPERTENSIONAHA.125.24916)
Supplement: Supplementary file 2 [file hyp-83-e24916-s002.pdf]

# Full unedited immunoblots

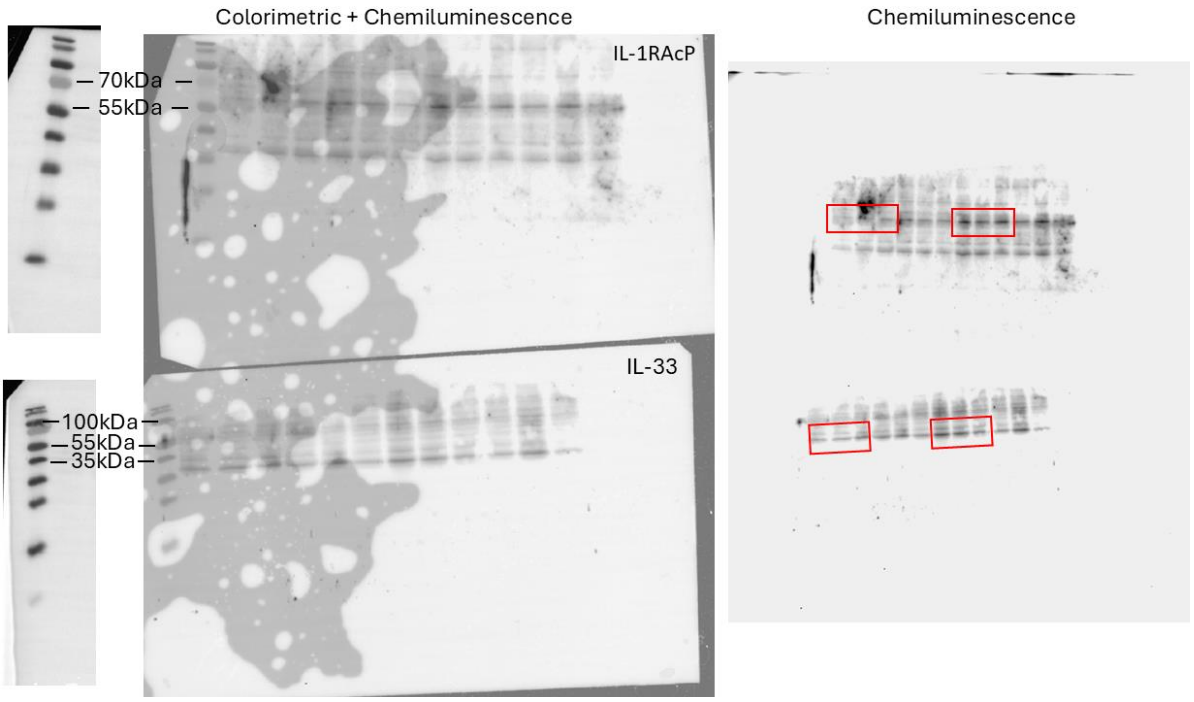


**Full unedited gel for Figure 1F.** Red square highlights the corresponding lanes of the blot in the original article

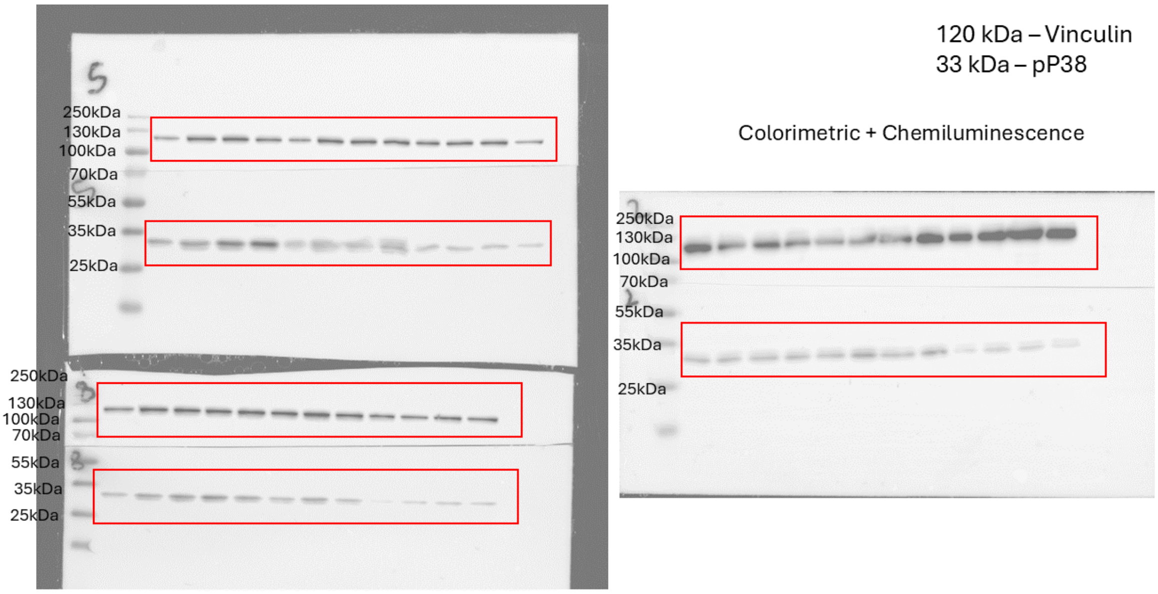


**Full unedited gel for Figure 4B**. Red square highlights the corresponding lanes of the blot in the original article

Colorimetric + Chemiluminescence

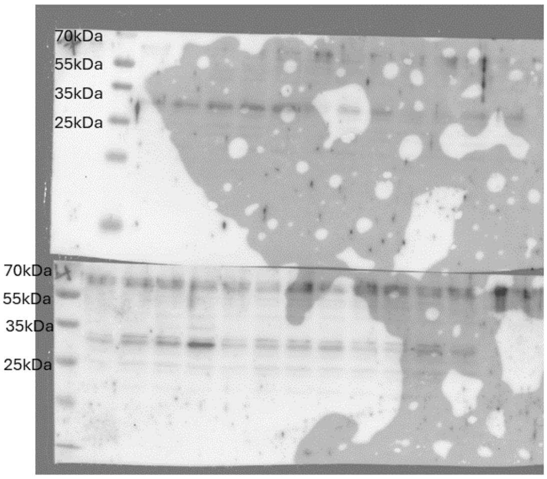


Chemiluminescence

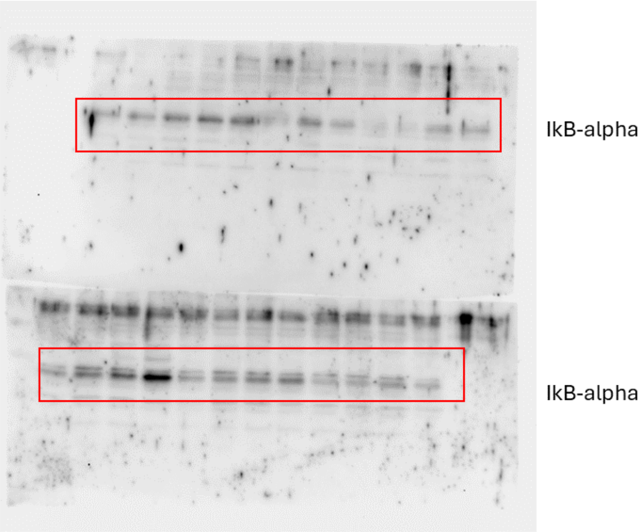


Colorimetric + Chemiluminescence

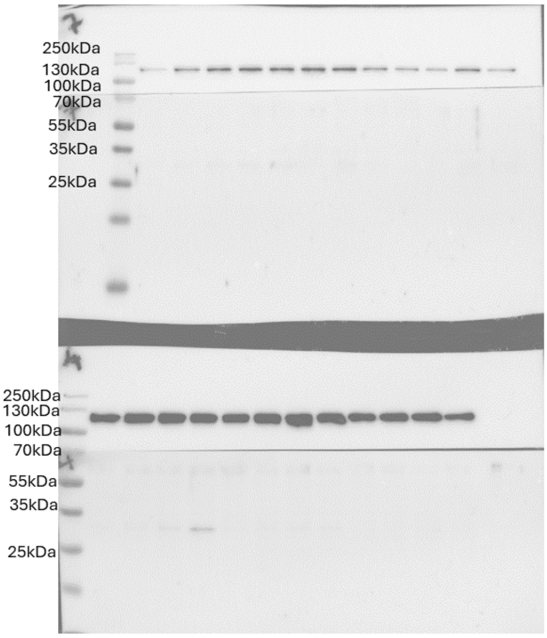


Chemiluminescence

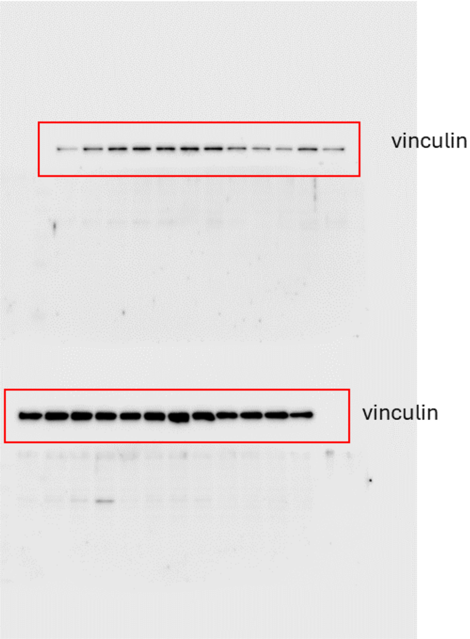

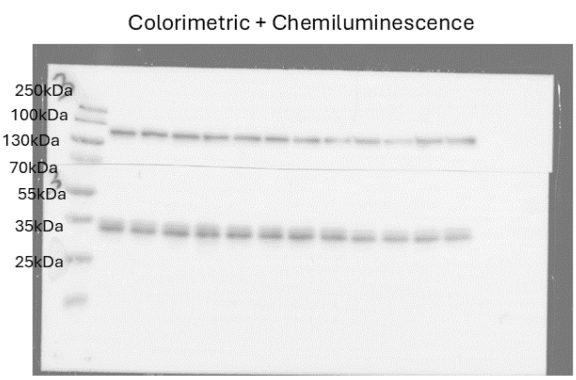


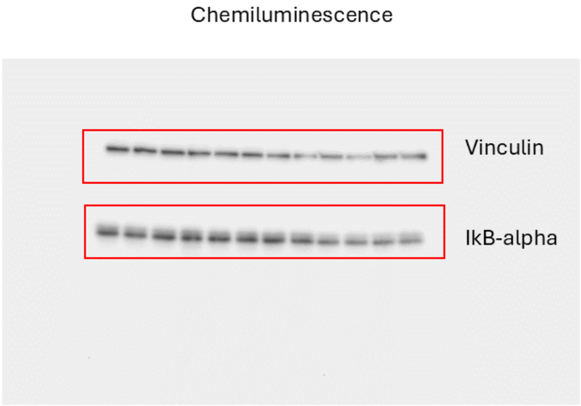


**Full unedited gels for Figure 4C.** Red square highlights the corresponding lanes of the blot in the original article
